# Wireless Modular Implantable Neural Device with One‐touch Magnetic Assembly for Versatile Neuromodulation

**DOI:** 10.1002/advs.202406576

**Published:** 2024-12-03

**Authors:** Inho Kang, John Bilbily, Choong Yeon Kim, Chuanqian Shi, Manish K. Madasu, Eun Young Jeong, Kyle E. Parker, Do A Kwon, Bum‐Joon Jung, Jae‐Soon Yang, Juhyun Lee, Noah D.L. Kabbaj, Wonhee Lee, Jun‐Bo Yoon, Ream Al‐Hasani, Jianliang Xiao, Jordan G. McCall, Jae‐Woong Jeong

**Affiliations:** ^1^ School of Electrical Engineering Korea Advanced Institute of Science and Technology (KAIST) Daejeon 34141 Republic of Korea; ^2^ Department of Anesthesiology Washington University in St. Louis St. Louis MO 63110 USA; ^3^ Department of Pharmaceutical and Administrative Sciences University of Health Sciences and Pharmacy in St. Louis St. Louis MO 63110 USA; ^4^ Center for Clinical Pharmacology University of Health Sciences and Pharmacy in St. Louis and Washington University School of Medicine St. Louis MO 63110 USA; ^5^ Washington University Pain Center Washington University in St. Louis St. Louis MO 63110 USA; ^6^ Department of Psychiatry Washington University in St. Louis St. Louis MO 63110 USA; ^7^ KAIST Information & Electronics Research Institute Daejeon 34141 Republic of Korea; ^8^ Department of Mechanical Engineering University of Colorado Boulder Boulder CO 80309 USA; ^9^ Department of Materials Science and Engineering KAIST Daejeon 34141 Republic of Korea; ^10^ Graduate School of Nanoscience and Technology KAIST Daejeon 34141 Republic of Korea; ^11^ Department of Physics KAIST Daejeon 34141 Republic of Korea; ^12^ Department of Bio and Brain Engineering KAIST Daejeon 34141 Republic of Korea; ^13^ KAIST Institute for Health Science and Technology Daejeon 34141 Republic of Korea

**Keywords:** magnetic assembly, modular, neural device, neuromodulation, wireless

## Abstract

Multimodal neural interfaces open new opportunities in brain research by enabling more sophisticated and systematic neural circuit dissection. Integrating complementary features across distinct functional domains, these multifunctional neural probes have greatly advanced the interrogation of complex neural circuitry. However, introducing multiple functionalities into a compact form factor for freely behaving animals presents substantial design hurdles that complicate the device or require more than one device. Moreover, fixed functionality poses challenges in meeting the dynamic needs of chronic neuroscience inquiry, such as replacing consumable parts like batteries or drugs. To address these limitations, the modular implantable neural device (MIND) is introduced with a one‐touch magnetic assembly mechanism. Leveraging the seamless exchange of neural interface modules such as optical stimulation, drug delivery, and electrical stimulation, MIND ensures functional adaptability, reusability, and scalability. The versatile design of MIND will facilitate brain research by enabling simplified access to multiple functional modalities as needed.

## Graphical abstract

The modular implantable neural device (MIND) features a one‐touch magnetic assembly mechanism for seamless exchange of neural interface modules, including optical stimulation, drug delivery, and electrical stimulation. Designed for functional adaptability, reusability, and scalability, MIND overcomes the challenges of fixed functionality neural devices, facilitating advanced, flexible neuroscience research in freely behaving animals with simplified access to multiple modalities.

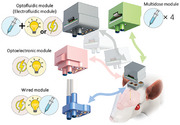

## 1 Introduction

Neuroscience has long sought to better understand how brains function. Recent breakthroughs in materials science and electrical engineering have enabled substantial progress in neural interfacing devices. These multifunctional and multimodal neural probes to dissect neural circuits and cellular signaling in the nervous system are becoming critical to advance our understanding of the mammalian brain due to their versatility and use in freely moving animals.

Conventional neural probes are typically confined to a single functional modality.^[^ ^1^, ^2^, ^3^, ^4^, ^5^, ^6^, ^7^, ^8^, ^9^, ^10^ ^]^ Despite the availability of a wide range of modalities, including electrical and optical stimulation, neuropharmacology, and electrical and optical neurophysiological recordings, the fixed and limited functionality of devices restricts the ability to test across functional domains within the same animal. To study the same circuits in different modalities, either multiple animals need to be used or traditional devices require substantial redesigning and refabrication, making multimodal investigation costly and time‐consuming. Furthermore, switching between different neural modalities or functionalities often requires multiple animals or multiple surgeries, which can increase variability and impact the neural system of interest, thereby reducing efficiency and replicability. To this end, an approach that seamlessly incorporates multiple functionalities within a single device can open a wide variety of opportunities for versatile investigation of neural circuit contributions to behavior.

Recent breakthroughs in materials and microfabrication techniques have enabled advanced, compact neural devices with multiple functionalities, including a combination of physiological recording, photostimulation, and fluid delivery.^[^ ^11^, ^12^, ^13^, ^14^, ^15^, ^16^, ^17^, ^18^, ^19^, ^20^ ^]^ These multifunctional approaches certainly hold great potential for driving forward basic neuroscience research and clinical interventions for neurological disorders. However, to accommodate each modality, devices have become increasingly complex, with control hardware and neural interfaces squeezed into a single compact platform. Such intricate and fixed designs compromise customization and functional scalability, posing challenges in meeting the evolving demands of neuroscience. In particular, chronic implants face limitations due to the need to replace consumable materials such as batteries or drugs (Table S1, Supporting Information).

To address these limitations, we introduce the Modular Implantable Neural Device (MIND). This device redefines neural interfacing capabilities with its functional adaptability, reusability, and scalability. MIND's modular design and one‐touch magnetic assembly mechanism enhance experimental flexibility by allowing the simple exchange of neural interface modalities such as electrical stimulation, photostimulation, and fluidic delivery. This device also minimizes disruptions to the animals during modality changes, such as anesthesia or subsequent surgeries. Here, we present the design and working principles of MIND and begin to explore its potential for brain research. With its modularity, multifunctionality, and design flexibility, MIND will facilitate multimodal investigation of brain function.

## 2 Results

### 2.1 Concept and Design of Modular Implantable Neural Device (MIND)

**Figure 1:**
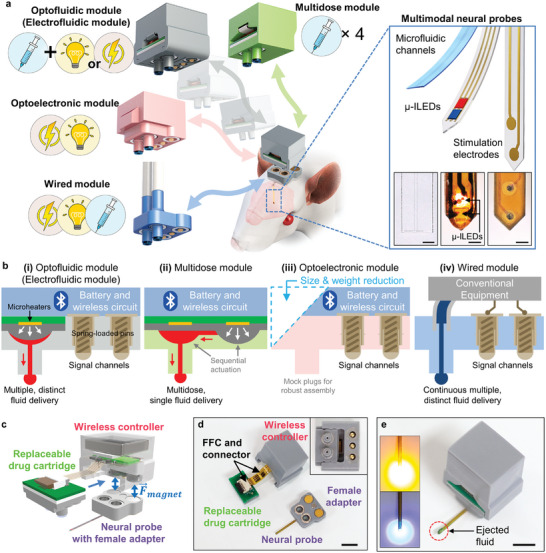
Wireless modular implantable neural device (MIND) for versatile neural interfaces. a) Conceptual illustration demonstrating the one‐touch magnetic assembly and disassembly of functional modalities (e.g., a fluidic, optical, electrical, or customizable combination thereof) within the implanted neural probe device. This magnetic plug‐and‐play approach facilitates rapid and seamless alternations in device functionality, power replenishment, and drug restocking in conscious, freely moving animals. The inset on the right offers an exploded view of a multimodal neural probe with capabilities of drug delivery (left), photostimulation (middle), and electrical stimulation (right), which can be selectively combined for specific applications (scale bars, 200 µm). b) Cross‐sectional views of various modules highlighting their functional designs. i) Optofluidic or electrofluidic module featuring two independent fluidic chambers for distinct fluid deliveries and two signal paths for optical or electrical stimulation control. ii) Multidose module capable of multiple deliveries of the same fluid through sequential activation of serially connected drug chambers. iii) Optoelectronic module providing electrical and/or photostimulation. iv) Wired module, fully compatible with conventional lab equipment (e.g., fluid pump, signal generator, etc.), capable of delivering fluid, photostimulation, and/or electrical stimulation. c,d) Schematic diagram (c), and optical image (d) highlighting modular components of wireless MIND, including a replaceable drug cartridge, a wireless control module, and a neural probe with a female adapter. The replaceable drug cartridge is powered and controlled by the wireless controller via a flexible flat cable (FFC). The inset in (d) demonstrates the plug‐and‐play assembly of a wireless control module and a replaceable drug cartridge facilitated by the bumpy structure. Scale bar, 5 mm. e) Optical image of the assembled device showcasing its versatile control capabilities for independent delivery of fluid and multiple wavelengths of light. Scale bar, 5 mm.

**Figure** **1**a illustrates the concept and design architecture of the modular implantable neural device (MIND). The MIND primarily consists of the implantable neural probe and the replaceable functional modules, each equipped with plug‐n‐play female and male adapters integrated with small magnets. The device exhibits modularity in two critical dimensions. First, the neural probes offer different modalities (e.g., fluidic, optical, and electrical) that can be selectively assembled to provide customized multimodal neural interfaces. Second, the design incorporates a user‐friendly one‐touch magnetic assembly/disassembly mechanism for diverse functional modules, allowing for easy adjustment and expansion of neuromodulation modalities. The range of functional modules includes but is not limited to, optofluidic modules (suitable for applications in optogenetics, pharmacology, and optopharmacology),^[^ ^21^, ^22^, ^23^, ^24^ ^]^ electrofluidic modules (employing precise electrical stimulation with the same fluidic mechanism as optofluidic modules),^[^ ^25^, ^26^, ^27^ ^]^ multidose modules (tailored for chronic pharmacological studies), optoelectronic modules (supporting optogenetics and electrical stimulation),^[^ ^28^, ^29^, ^30^ ^]^ and wired modules (enabling seamless integration with conventional neuroscience equipment). When the neural probe is chronically implanted into the brain, the female adapter is securely affixed to the cranium. This stable foundation enables these functional modules to be effortlessly attached and easily exchanged through magnetic assembly to the female adapter. Together, these features allow for the creation of customized functional neural interfaces for specific research requirements.

This novel modular assembly of the MIND using magnetic force offers several compelling advantages in neuroscience research involving live animals. First, the physical pressure required for the assembly of a functional module can be considered negligible due to the mutual attraction of magnet counterparts (i.e., assembly force ≈0 N). This easy exchange allows for the rapid replacement of a module (<3 s) with minimal disturbance to the natural behavior of animals while also eliminating the need for anesthesia during the process. Additionally, using magnets as an active source of assembly force ensures a robust and stable module connection, thereby providing a reliable platform for in vivo animal experiments. This stability is crucial for the accuracy and consistency of research findings. Furthermore, this modular assembly facilitates a wide range of studies across various neuromodulation modalities. Researchers can simply plug in a different module without the need to create an entirely new device with distinct capabilities, subject animals to multiple surgeries, or use multiple animals to test hypotheses across modalities. This versatility streamlines the research process, fostering straightforward testing of diverse neural circuit functional contributions to behavior through adaptable experimentation.

Figure 1b depicts the design and mechanisms of various functional modules developed in this study (for device specifications, refer to Table S2, Supporting Information). The optofluidic module (Figure 1b(i)) serves the purpose of selectively delivering multiple, distinct fluids and light with different wavelengths (here, 470 and 589 nm, but adaptable to other µ‐ILEDs) to the brain. In this module, fluid delivery is enabled by thermally‐actuated pumps leveraging thermally expandable polymer, as we previously demonstrated.^[^ ^16^ ^]^ The electrofluidic module possesses the same structural design as the optofluidic module, differing only in the internal circuitry. This circuitry facilitates signal modulation specifically tailored for electrical stimulation, as opposed to optical stimulation. The multidose module (Figure 1b(ii)) offers the sequential delivery of multiple fluids through the actuation of serially‐connected, thermally‐actuated micropumps. This feature can be useful for chronic in vivo pharmacology experiments involving repeated drug infusions with minimal disruption to animal behavior. The optoelectronic module (Figure 1b(iii)) is designed to provide a selective combination of optical and electrical stimulation. Remarkably, this module is 36% smaller in size and 53% lighter than the optofluidic module because there is no drug cartridge. The wired module (Figure 1b(iv)) enhances the versatility of MIND by allowing the integration of conventional equipment, including a fluid pump for drug delivery and a signal generator for optical and electrical stimulation (Figure S1, Supporting Information). Importantly, all modules are lightweight (ranging from 0.86 to 1.56 g) and compact (dimensions ranging from 670 to 1050 mm^3^), rendering them well‐suited for behavior experiments in small animals (Figure S2, Supporting Information).^[^ ^12^ ^]^

Figure 1c,d, highlight the modularity of the MIND's components and the straightforward nature of their assembly and disassembly, using the optofluidic module as a representative example. The device design facilitates repeated fluid infusion and battery charging, as well as exchanging the functional module. This specific optofluidic module is equipped with a replaceable drug cartridge and a wireless controller (Figure 1c,d) that are electrically interconnected via a flexible flat cable (FFC) and mechanically secured using the bumpy structures shown in Figure 1d, inset, and Figure S3 (Supporting Information). Recharging the battery and refilling drugs is a simple process: the functional module is removed from the neural probe part, the lithium polymer (LiPo) battery (GMB401010, 28 mAh, PowerStream Technology) is charged, and a new drug cartridge integrated into the wireless controller is replaced. Here, the LiPo battery that is housed within the wireless controller can be recharged by connecting the controller to a custom‐designed battery charger without the need to extract the battery from the device, enhancing user convenience (Figure S4, Supporting Information). Upon reassembling the replenished functional module into the probe part, the MIND can, as shown in Figure 1e, wirelessly and selectively control each modality (fluid delivery or optical stimulation, in this example) in response to commands transmitted through a custom‐designed smartphone app based on Bluetooth Low Energy (BLE) communication (Figure S5, Supporting Information; working distance ≈10 m).

### 2.2 Mechanical Characterization of Magnetic Assembly Facilitating Robust Fluidic and Electrical Connections

**Figure 2:**
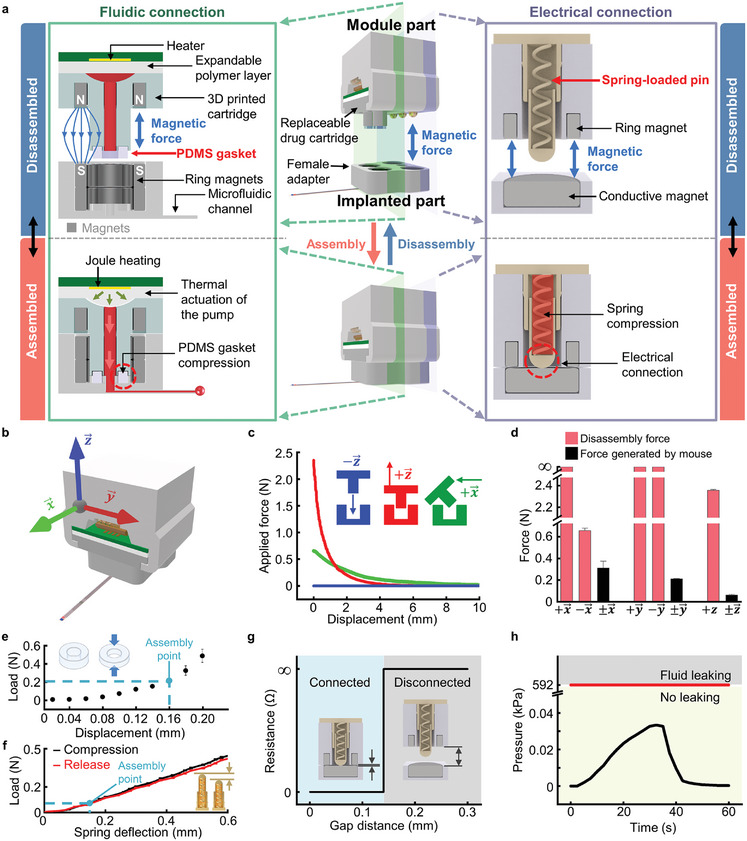
Mechanical characteristics of magnetic assembly interface enforcing robust fluidic and electrical connection. a) Illustration of the magnetic assembly and disassembly with cross‐sectional views detailing the mechanisms of fluidic and electrical connections. Fluidic connections are established through the compression of PDMS gaskets, while electrical connections are secured by the compression of spring‐loaded pins, both enabled by strong magnetic attraction between magnets in the female adapter of the implanted part and the replaceable module part. b) Schematic diagram defining the axis of the force applied to MIND. c) Assembly (blue) and disassembly forces (red and black) as functions of displacement in the directions shown in each of the inset figures. No external force is necessary for assembly due to magnetic attraction (blue line), whereas certain levels of force need to be applied for disassembly (red and black line). Note that the device is designed to be disassembled only in the ‐x and +z directions. d) Comparison between the force required to disassemble and the force generated by the movement of a mouse.^[^ ^28^ ^]^ The forces generated by the mouse are significantly lower than the required disassembly force, ensuring robust assembly. e,f) The restoring forces of a PDMS gasket (e) and a spring‐loaded pin (f) along the z‐axis. Both restoring forces are much lower than the force required for disassembly, causing negligible influence on the magnetic assembly. g) Resistance of an electrical connector at the magnetic assembly interface as a function of gap distance between the replaceable module part and the implanted part. h) Comparison of the critical pressure threshold for fluid leakage at the magnetic assembly interface (red line) and fluid pressure generated during fluid delivery (black line). The magnetic assembly resists leakage, as the maximum fluid pressure is 17,760 times lower than the critical pressure threshold for fluid leakage.

**Figure** **2**a illustrates the working mechanisms governing the fluidic and electrical connections between the two primary components of MIND, which are the implanted probe part and the functional module part, enabling fluid delivery, and optical, and electrical stimulation. These connections rely on magnetic attraction forces and a plug‐n‐play structural assembly involving male and female adapters on the respective MIND components. For fluid delivery, the microfluidic male adapters, incorporating magnets from the replaceable drug cartridge in the module part, seamlessly connect with the female adapters housing two ring‐shaped magnets (3 mm‐outer diameter (OD), 2 mm‐inner diameter (ID), 1 mm‐thick), facilitating fluid delivery throughout the microfluidic neural probe (Figure 2a, left). During this connection, soft polydimethylsiloxane (PDMS; E (Young's modulus) ≈1 MPa) gaskets (1.5 mm‐OD, 0.7 mm‐ID, 0.55 mm‐thick) affixed to the male adapters compress due to the mutual attraction of the magnets, ensuring hermetic sealing and preventing fluid leakage when the pump is engaged. The electrical connection employs a one‐touch magnetic assembly established through compressing three conductive spring‐loaded pins (σ (conductivity) ≈1.5 × 10^7^ S m^−1^) against three disk‐shaped, conductive magnets (each side: 3 mm‐diameter, 1 mm‐thick; center: 1.5 mm‐diameter, 1 mm‐thick) integrated within the implanted probe part (Figure 2a, right). The exposed tips of the spring‐loaded pins, extending outward (≈0.14 mm), undergo compression via magnetic attraction force during the electrical connection. This design takes advantage of the spring‐loaded pins’ ongoing potential outward expansion, leading to a reliable electrical connection between the two components.

To assess the robustness of the magnetic assembly for use in animal experiments, we examined the forces required for the assembly and disassembly of the functional module and implanted parts along different axes (Figure 2b). No external force (i.e., F ≈0 N) is needed during MIND assembly due to mutual magnetic attraction along the −z direction (Figure 2c, blue line). However, a substantially high force (≈2.35 N) is necessary for disassembly in the +z direction to overcome magnetic attraction (Figure 2c, red line), mitigating concerns about unintended removal of the module during freely moving behavior. When module replacement is necessary, disassembly can be relatively easily achieved by applying a small force (≈0.66 N) in the horizontal direction (i.e., −x direction; Figure 2c, green line). It is important to note that MIND is designed to prevent unintended disassembly in all directions except for +z and −x directions to ensure reliable device operation during the natural behavior of animals (Figure S6 and see Note S1, Supporting Information). However, in both +z and −x directions, accidental disassembly by natural movements in small animals, such as mice, is not a concern due to insufficient force exerted by their typical motions (lacking in disassembly force by 110% in −x direction and by 3700% in +z direction).^[^ ^28^ ^]^ This feature ensures reliable mechanical, electrical, and fluidic connections required for operation in freely behaving animals (Figure 2d).

We next further analyzed the robustness of MIND's magnetic assembly by considering the restoring forces produced by the PDMS gaskets and spring‐loaded pins. When the MIND parts are assembled, PDMS gaskets and spring‐loaded pins are compressed by 0.16 mm and 0.14 mm, respectively, resulting in reactive forces of ≈0.20 and ≈0.06 N (Figure 2e,f). Considering the presence of two gaskets and three spring‐loaded pins within the optofluidic module, the total restoring force is ≈0.58 N, less than one‐fourth of the magnetic attraction force between the two MIND parts (≈2.35 N). This indicates that the magnetic assembly can be stably maintained despite the restoring force, ensuring reliable electrical and fluid connections. Simultaneously, thanks to the restoring force of spring‐loaded pins, a stable electrical connection can be maintained even with slight detachment (≈0.14 mm) of the functional module from the implanted part (Figure 2g). Furthermore, this magnetic assembly demonstrated reliable electrical and fluidic connectivity over 1000 cycles of assembly and disassembly, underscoring its reliability for long‐term use (Figure S7, Supporting Information).

To verify the leakage‐free fluidic connection between the two MIND parts, we evaluated internal fluid pressure during pumping and compared it to the pressure required to cause fluid leakage. According to finite element analysis (FEA) simulation (Figure S8, Supporting Information), while a drug reservoir (0.5 µL) is actuated for 30 s, internal fluid pressure peaks at 0.033 kPa. This pressure is substantially smaller (17,760 times lower) than the critical leaking pressure of 592 kPa required to breach the hermetic sealing of the fluidic connection (Figure 2h and see Note S2 for details, Supporting Information), ensuring a robust fluidic connection between the module and the implanted probe parts. These results collectively demonstrate that the magnetic assembly of the MIND facilitates robust fluidic and electrical connections, which are essential for use in freely moving animals.

### 2.3 Optical, Electrical, and Fluidic Characteristics of MIND Modalities

**Figure 3:**
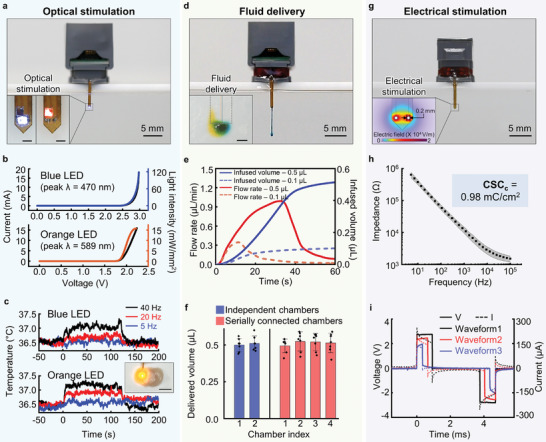
Characteristics of optical, electrical, and fluidic modality. a–c) Photostimulation. a) Optical image of a device demonstrating multi‐color (blue and orange) μ‐ILED operation. The insets show independent control of µ‐ILEDs (Scale bars, 300 µm). b) Current and optical intensity of blue (top) and orange (bottom) µ‐ILEDs as a function of voltage. c) Temperature increases of brain tissue during photostimulation at different pulse frequencies (5, 20, and 40 Hz; with a 10 ms pulse width). These changes are measured using an infrared camera after inserting the probe into brain tissue at a depth of 1 mm. The inset offers an optical image of the optogenetic probe inserted into the brain tissue. d–f) Fluid delivery. d) Optical image of a device delivering an aqueous solution with blue dye into water. The inset shows delivery of multiple, distinct fluids (yellow and blue) into brain phantom (0.6 wt% agarose gel). e) Flow rate and infused volume over time following pump actuation at *t* = 0 s. 3D printed drug cartridges with 0.5 µL (solid lines) and 0.1 µL reservoirs (dotted lines) are examined, demonstrating the ability to customize doses using 3D printing technology. f) Volume comparison of delivered fluid from two different drug cartridges designed for the target delivery volume of 0.5 µL. Blue bars represent the delivered volume by the optofluidic or electro‐fluidic module with independent chambers, and red bars represent the result from the multidose module with serially connected chambers. g–i) Electrical stimulation. g) Photograph of a device with stimulation electrodes on the probe. The inset represents the simulated electrical field distribution created between the pair of electrodes with an applied voltage of 3 V. h) the Electrical impedance of a stimulation electrode as a function of frequency. i) Demonstration of the capability of electrical stimulation intensity, frequency, and pulse width modulation.

**Figure** **3** presents various assembly configurations of MIND functional modules, each highlighting distinct modality characteristics. One method for optogenetic neuromodulation is assembling an optofluidic or optoelectronic module into an optogenetic probe (Figure 3a). This optogenetic probe can precisely deliver light, either simultaneously or selectively, in two different wavelengths to the brain region of interest. Here, we do so by incorporating two distinct types of μ‐ILEDs (TR2227, Cree for 470 nm blue light and TCE10‐589, Three Five Materials for 589 nm orange light). The integrated blue and orange μ‐ILEDs can emit light with maximum intensities of 120 and 16 mW mm^−2^, respectively (Figure 3b), surpassing the thresholds required for the activation of channelrhodopsin (ChR2; 1 mW mm^−2^)^[^ ^29^, ^30^, ^31^ ^]^ and halorhodopsin (NpHR; 7 mW mm^−2^).^[^ ^29^ ^]^ Furthermore, we show that optical stimulation using MIND is thermally compatible with brain tissue. Operating both blue and orange μ‐ILEDs under various conditions (5, 20, and 40 Hz, 10 ms pulse width) below a temperature‐maintained (36.5 °C) 1 mm thick‐brain slice, the surface temperature of the brain slice increased by less than 1 °C in all cases (Figure 3c). This indicates that during MIND's optical stimulation temperature remains lower than the maximum allowed for the thermally safe operation of implantable devices.^[^ ^12^, ^13^, ^16^, ^31^, ^32^, ^33^, ^34^ ^]^

Fluid delivery for in vivo pharmacology can be achieved through the combination of an optofluidic, electrofluidic, or multidose module with a probe that integrates a microfluidic channel (Figure 3d). The drug cartridge within the optofluidic or electrofluidic module contains two reservoirs, each connected to different microfluidic channels, enabling selective delivery of two distinct types of fluids (Figure 3d, inset). Drug cartridges can be rapidly customized with various reservoir volumes using 3D printing technology based on high‐resolution stereolithography. For example, we designed drug cartridges with different infusion volumes of 0.1 and 0.5 µL for distinct pharmacological approaches, as demonstrated in Figure 3e. During actuation, the 0.1 µL chamber can dispense the fluid contained within it in 10 s, generating a fluid pressure of up to 0.0012 kPa, while the 0.5 µL chamber can do so in 30 s, resulting in a maximum pressure of 0.033 kPa. Fluid pressures in this range have a negligible impact on the brain.^[^ ^16^ ^]^ Additionally, 3D‐printed drug cartridges feature uniform dosage delivery. Cartridges consisting of two independent chambers with 0.5 µL capacity show a relative standard deviation (RSD) of 9.2%, and cartridges with four serially connected chambers show an RSD of 11.5% (Figure 3f). These low RSD values confirm the reliable and consistent reproducibility of the 3D‐printed drug cartridges. The modular interchangeability and customizable dosages of the drug cartridges with high reproducibility enable versatile applications for chronic in vivo experiments.

Electrical microstimulation can also be performed by assembling an optoelectronic or electrofluidic module into an electrical stimulation probe part (Figure 3g). The electrodes of the electrical stimulation probes are plated with gold (Au; 4 µm‐thick) and platinum (Pt; <1 µm‐thick), resulting in a low contact impedance of 58 kΩ at 100 Hz (Figure 3h), enabling neural activation through electrical impulses at low output voltages (<3 V). The optoelectronic module can generate diverse biphasic waveforms by modulating intensity, frequency, and pulse width (Figure 3i). The output terminals of a Bluetooth Low Energy System‐on‐Chip (BLE SoC; EYSHSNZWZ, Taiyo Yuden) are consolidated into a single port by connecting them with different resistors in parallel. Using a custom‐designed smartphone app, researchers can wirelessly adjust the intensity of electrical stimulation in three discrete steps (2.9, 2.5, and 2.1 V). This is achieved by selecting an appropriate output terminal among the output pins, each connected to a resistor with a different resistance, to tailor the stimulation intensity to specific needs. Additionally, our MIND system exhibits high flexibility in modulating stimulation frequency (ranging from DC to 1 kHz) and pulse width (0–50% duty cycle) by adjusting relevant parameters on the smartphone app's user interface.

The modular design of MIND, coupled with its simple plug‐n‐play architecture, facilitates the straightforward exchange of the optical, electrical, fluidic, and any combination of these modalities for versatile neuromodulation. We next demonstrate this flexible application of device function in freely moving animals.

### 2.4 In Vivo Validation of Individual Optical and Fluidic Modalities of MIND

**Figure 4:**
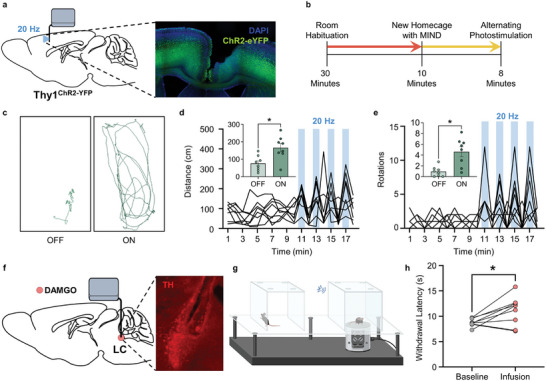
In vivo validation of optoelectronic and fluidic modules. a–e) Validation of in vivo photostimulation using locomotor behavior. a) Left, sagittal view of the mouse brain schematic showing implantation of optoelectronic MIND module into the secondary motor cortex of Thy1ChR2 mice. Right, fluorescent micrograph showing probe placement and ChR2‐YFP expression. b) Calendar of experimental protocol measuring locomotion in a novel homecage. c) Representative traces of animal position during the 10th and 11th min show the optoelectronic MIND module increases the distance traveled in a photostimulation‐dependent manner, which is quantified in (d). The inset in (d) shows the total distance traveled with and without photostimulation (Paired *t*‐test, *p* = 0.0324, *n* = 8). e) photostimulation also increases rotations. The inset shows total rotations with and without photostimulation (Paired *t*‐test, *p* = 0.011, *n* = 8). f–h) Validation of in vivo fluid delivery using pharmacologically‐induced antinociception. f) Left, sagittal view of the mouse brain schematic showing implantation of fluidic MIND module into the mouse LC. Right, immunofluorescent image showing probe placement in the LC, identified by tyrosine hydroxylase (TH) immunoreactivity. g) Cartoon of multiple mice in the enclosed thermal plantar assay with BLE‐mediated wireless drug delivery through the fluidic MIND module. h) Infusion of the mu‐opioid agonist, DAMGO, into the LC produces thermal antinociception (Paired *t*‐test, *p* = 0.0260, *n* = 9).

A key advantage of MIND is its flexibility in design to access distinct modes of neuromodulation for in vivo neuroscience in freely moving animals. To demonstrate the MIND photostimulation functionality, we used mice expressing ChR2 under the Thy1 promoter.^[^ ^35^ ^]^ Here we implanted the optoelectronic MIND probe in the right secondary motor cortex (M2) (**Figure** **4**a). After eight weeks of chronic implantation, we then used the one‐touch assembly mechanism to attach the optoelectronic module while the animal freely explored a new home cage environment (Figure 4b). Then, as we and others have before,^[^ ^16^, ^35^ ^]^ we photostimulated M2 at 20 Hz using the 470 nm µ‐ILED. This MIND‐mediated photostimulation significantly increased both the distance traveled and the total number of rotations (Figure 4c–e) of mice freely moving in a normal home cage environment, clearly demonstrating the successful deployment of the optical stimulation module.

We next sought to test the wireless drug delivery function in an enclosed space. Most evoked sensory testing in preclinical animal models takes place in fully enclosed boxes with multiple animals separated by thin barriers, making wireless neuromodulation especially convenient for increasing throughput. Here, we turned to the thermal plantar assay to test the fluidic MIND module. Due to its well‐established role in modulating nociception, we implanted fluidic MIND modules into the locus coeruleus (LC) of a new cohort of mice (Figure 4f). While the LC can provide bidirectional control of nociception,^[^ ^36^, ^37^ ^]^ we recently demonstrated that inhibition of the LC in naïve mice is antinociceptive.^[^ ^38^, ^39^ ^]^ Endogenous opioid systems provide powerful inhibition of LC neurons. Direct mu‐opioid receptor (MOR) agonism in the LC robustly hyperpolarizes these neurons and intra‐LC infusions of the MOR agonist [D‐Ala2, N‐MePhe4, Gly‐ol]‐enkephalin (DAMGO) or the endogenous opioid peptide met‐enkephalin is antinociceptive.^[^ ^38^, ^39^, ^40^, ^41^, ^42^, ^43^, ^44^, ^45^ ^]^ Here, seven weeks after chronic MIND implantation, we used the entirely enclosed thermal plantar assay to test the wireless delivery of DAMGO into the LC (Figure 4g). As expected, intra‐LC DAMGO increased the latency to withdraw from a noxious thermal stimulus on the ipsilateral hind paw (Figure 4h), indicating successful wireless MIND‐mediated intracranial fluid delivery through an enclosure.

### 2.5 Repeated Wireless Electrical Stimulation and Pharmacology in Awake, Behaving Mice

**Figure 5:**
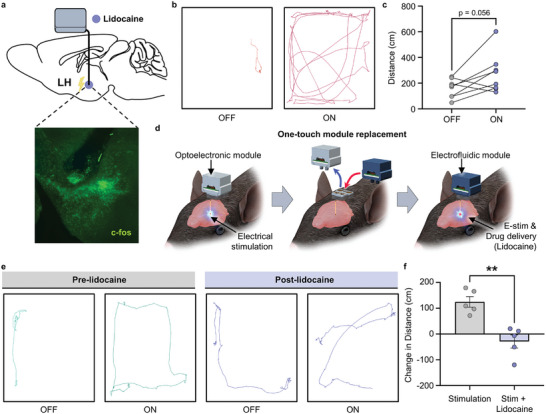
One‐touch exchange from optoelectronic to electrofluidic module in freely moving mice. a–f) Validation of in vivo electrical microstimulation with concurrent drug delivery within the same subject using locomotor behavior. a) Top, sagittal view of the mouse brain schematic showing implantation of electrofluidic MIND module into the mouse LH. Bottom, an immunofluorescent image showing probe placement in the LH and electrical stimulation‐evoked c‐fos immunoreactivity. b) Representative traces of animal position during the first electrical stimulation. c) The same electrical stimulation parameters (130 Hz, 2%, duty cycle) delivered to all mice increased locomotion of C57BL/6J mice in the open‐field box (Paired *t*‐test, *p* = 0.0561, *n* = 8). d) Cartoon showing the one‐touch module replacement approach for a subsequent day, in which animals that had more than a two‐fold increase in distance traveled were selected for the one‐touch multimodal experiment with lidocaine infusion. e) Representative traces of animal position before and during electrical stimulation, with and without intra‐LH lidocaine delivery. f) Here, the increase in distance traveled with electrical stimulation was blocked with 2% lidocaine infusion following one‐touch exchange to the electro fluidic module (Paired *t*‐test *p* = 0.0034, *n* = 5).

One of the primary advantages of MIND is its ability to swiftly and easily switch modules and approaches for neuromodulation. In particular, the one‐touch magnetic assembly enables a seamless transition from one modality to another. To test this design feature in freely moving animals, we designed an experiment that began with electrical stimulation through the optoelectronic module and concluded with an electrofluidic module for combined electrical and pharmacological neuromodulation. In this experiment, after 9–12 weeks of chronic implantation, we first tested the in vivo electrical microstimulation capabilities of MIND by implanting an electrofluidic probe into the right lateral hypothalamus (LH), an area known to promote arousal and increase locomotor activity (**Figure** **5**a).^[^ ^7^, ^46^ ^]^ We first used electrical stimulation alone to increase the distance traveled in an open field (Figure 5b,c). The following day, we adjusted the electrical stimulation parameters to achieve at least a two‐fold increase in locomotor activity in each mouse (Figure S9, Supporting Information). Finally, to demonstrate the ease of the one‐touch multimodal function using MIND, we next combined electrical microstimulation and fluidic capabilities in vivo. Here, mice that demonstrated a two‐fold or greater increase in locomotion were now electrically stimulated again. Following this initial stimulation, the optoelectronic module was switched to an electrofluidic module loaded with the voltage‐gated sodium channel blocker lidocaine (Figure 5d). Following infusion, lidocaine occluded the effect of electrically stimulating the LH (Figure 5e,f). These results demonstrate that the modular design of MIND, coupled with its simple plug‐n‐play architecture, facilitates the straightforward exchange of the optical, electrical, fluidic, and any combination of these modalities for versatile neuromodulation.

## 3 Discussion

The modular neural device we introduce here, MIND, offers functional reconfiguration through seamless control‐module exchange using one‐touch magnetic assembly. MIND's modular architecture allows researchers to effortlessly interchange various modules, such as fluid delivery, photostimulation, electrical stimulation, and any such combination. This versatility facilitates easy reconfiguration of experimental setups, enabling researchers to address diverse neuroscience questions by allowing for a comprehensive investigation of neural processes without the need for additional animals in studies with different modalities. MIND's ability to rapidly (module replacement time < 3 s) and effortlessly (assembly force ≈0 N) replace the modules further enhances its utility, particularly for chronic in vivo experiments with freely behaving animals. This feature ensures easy restoration of power or drug supply and eliminates the need for anesthesia during these processes, minimizing disturbance to the natural behavior of animals. Moreover, MIND's modular architecture significantly reduces both the time and costs associated with new tool development. Unlike conventional systems that require complete replacement in case of component damage or malfunction, MIND allows for efficient repair through the replacement of only the affected component, helping to ensure smooth operation and maintenance. This latter feature may help extend the lifetime of chronically implanted wireless devices. In this study, devices were implanted for long periods of time before behavioral experiments (i.e., 7–12 weeks). This approach ensured that the in vivo data presented here represents functionality across MIND modules after long periods of exposure to brain tissue and endogenous immune responses.^[^ ^12^, ^34^, ^47^ ^]^ Other than the within‐subject, repeated electrofluidic experiment (Figure 5), we did not directly test the long‐term repeated use of these devices. However, our data demonstrating intact connections for over 1000 cycles of assembly and disassembly combined with published work testing repeated functionality of similar electrical microstimulation interfaces^[^ ^7^ ^]^ suggests these devices will reliably function for at least a few months following implantation, and possibly much longer. Chronically implanted, modular neural devices will enable long‐term studies necessary to evaluate circuit function across meaningful timescales for the slow‐evolving neural adaptations that occur during aging, neurodegenerative diseases, depression, chronic pain, and other important clinical concerns. Such chronic functionality is a clear necessity for any device to translate to clinical use. Here, we demonstrated these features using a variety of proof‐of‐principle in vivo experiments with freely moving mice. These experiments demonstrate the versatility of the MIND in accessing distinct functional modalities within‐ and across experimental designs. In particular, MIND carries all of the previously established benefits of wireless, BLE‐enabled devices for selective and scalable control of neural circuits.

With its versatility and broad applications demonstrated through a series of animal studies, MIND shows immense potential in advancing behavioral neuroscience. Its adaptability and user‐friendly design offer unique opportunities for researchers to dissect neural circuits with enhanced precision and efficiency. While our current studies have focused on functional variation for neuromodulation, envisioning MIND's evolution to incorporate sensing modalities such as electrophysiological, optical, and biofluid sampling into the available toolset promises a transformative leap in our ability to dynamically observe brain activities in freely behaving animals. MIND will help deepen our understanding of the brain, reshaping the landscape of exploration and discovery in neuroscience studies.

## 4 Experimental Section

### Fabrication of Multimodal Neural Probes

The multimodal neural probes were fabricated by stacking individual neural probes with distinct modalities: i) electrical stimulation, ii) optical stimulation, and iii) drug delivery. The optical or electrical stimulation probes were manufactured by a commercial vendor (PCBWay). The flexible printed circuit board (FPCB) probes feature Cu/Au electrode layers (each 18 µm‐thick) on both sides of a polyimide substrate, resulting in a total probe thickness of 100 µm. Electrical stimulation probes were further coated with gold (≈4 µm‐thick) and platinum (<1 µm‐thick) on their electrodes. Optical stimulation probes consisted of a blue μ‐ILED (TR2227, CREE; 470 nm) and an orange μ‐ILED (TCE10‐589, III‐V MATERIALS; 589 nm) soldered onto the tip of the FPCB electrodes, coated with 7 µm‐thick Parylene C for biocompatibility and water‐resistance.^[^ ^48^ ^]^ Microfluidic probes for drug delivery were fabricated using PDMS through soft lithography processes, as presented in our previous work.^[^ ^49^ ^]^ Each probe could be selectively stacked to construct a multimodal neural probe using double‐sided silicone adhesive (ARClear 8932EE, Adhesive Research), as per the experimental needs.

### Fabrication of the Neural Probe with a Female Adapter

The female adapter facilitated a one‐touch magnetic assembly that connected neural probes with wireless control modules (Figure S10a, Supporting Information). After designing with Autodesk Inventor software (Autodesk), its body was 3D‐printed. Subsequently, ring magnets (R0525‐10, SuperMagnetMan) and conductive disk magnets (D1005B‐10 TiN, SuperMagnetMan) were inserted into the 3D‐printed body. After applying photopolymer to the inserted magnets, it was left to rest for ≈2 min to fill gaps before solidifying in a 7 min UV‐curing process. To assemble multimodal neural probes with a female adapter, conductive epoxy (8331, MG Chemicals) could be used to connect either electrical or optical stimulation probes with the conductive magnets of the female adapter, which function as electrodes for electrical connectors with a wireless control module. Additionally, the microfluidic channel and the female adapter were attached using double‐sided silicone adhesive.

### Design and Fabrication of Functional Wireless Modules with Male Adapters

Functional wireless modules deliver targeted neuromodulation signals and/or drugs to neural probes. The bottom of these modules formed a male adapter corresponding to the female adapter for either structural assembly (in the case of the optoelectronic module) or for both structural and fluidic connection (in the case of optofluidic, electrofluidic, and multidose modules). These modules include i) an electrical circuit with a flexible flat cable (FFC) cable for connecting to the drug cartridge and battery charger, ii) a 3D‐printed encapsulation with embedded magnets for magnetic assembly, and iii) electrical connection ports with spring‐loaded pins for a robust electrical connection to the female adapter part (Figure S10b, Supporting Information). Electronic components, including a voltage regulator (NCP114AMX330TCG, Onsemi), MOSFETs (RV1C001ZP, Rohm) for actuation switching, and BLE SoC (EYSHSNZWZ, Taiyo Yuden) for wireless control, were soldered onto the top layer of the FPCB (Figures S11 and S12(i), Supporting Information). These FPCBs were custom manufactured by a commercial vendor (PCBWay) after being designed using Altium Designer software (Altium). Additionally, a lithium‐polymer (LiPo) battery (GMB401010, GMBattery; 10 × 9 × 4 mm^3^, 28 mAh, 0.65 g) and three spring‐loaded pins (0900‐2‐57‐20‐72‐14‐11‐0, Mill‐Max Manufacturing Corporation) for electrical connection were attached to the bottom layer of the FPCB using solder paste (SMDLTLFP10T5, CHIPQUIK) and conductive epoxy (8331, MG Chemicals) (Figure S12(ii), Supporting Information). Subsequently, the wings of the FPCB, including the FFC, were bent at 90° (Figures S12(iii), S12 (iv), Supporting Information). This allowed easy insertion of the entire circuit into the 3D‐printed encapsulation (Core 530, B9Creations), where two ring‐shaped neodymium magnets (R0525‐10, SuperMagnetMan) were installed at its bottom (Figure S12(v), Supporting Information). After enclosing the top opening with a 3D‐printed cover, photopolymer (B9R‐8‐HD‐SLATE, B9Creations) was applied and UV‐cured for 5 min to fully seal the encapsulation, leaving only the FFC and the spring‐loaded pins exposed for connection to the drug cartridge and the female adapter, respectively (Figure S12(vi), Supporting Information).

### Fabrication of Replaceable Drug Cartridges

The replaceable drug cartridge consists of i) a drug chamber with the male adapter for fluidic connection to the female adapter, ii) resistive heaters for efficient Joule heating, and iii) a thermally expandable polymer layer that expels fluid from the drug chamber through expansion induced by Joule heating (Figure S10C, Supporting Information). The drug chamber was 3D printed following the CAD design created using Autodesk Inventor software (Autodesk). Also, PDMS gaskets were fabricated by casting a mixture of elastomer and the curing agent of PDMS (SYLGARD 184, Dow Corning) at a 10:1 ratio into a 3D‐printed mold. After installing two ring‐shaped magnets (R0525‐10, SuperMagnetMan) onto the drug chamber to facilitate magnetic force, the PDMS gaskets were bonded to the ends of two pillar structures within the drug chamber using silicone adhesive (ARClear 8932EE, Adhesive Research).

The resistive heaters were patterned using conventional photolithography and a lift‐off process on the top layer of a commercially produced FR‐4 double‐layer PCB, followed by the deposition of Cr (10 nm)/Au (180 nm) (Figure S13, Supporting Information) through electron beam evaporation. After that, Parylene C (10 µm‐thick) was conformally coated onto the surface of the actuator‐patterned FR‐4 PCB through a chemical vapor deposition process, which serves as a protective layer to prevent damage upon detachment of the expandable layer, enabling reuse. Subsequently, an FFC connector (FH34SRJ‐4S, Hirose Electric) for electrical connection with the control module was soldered onto the bottom layer of the FR‐4 PCB to facilitate electrical connection.

Lastly, PDMS (elastomer:curing agent ratio = 10:1) and expandable microspheres (Expancel 031 DU 40, AkzoNobel) were mixed in a 2:1 ratio and spin‐coated onto a polycarbonate board at 500 rpm for 30 s. This expandable layer was then cured in an oven at 60 °C for 12 h, resulting in a thermally expandable layer (400 µm‐thick), which was subsequently transferred onto the heaters of the FR‐4 PCB. As the final step, a 3D‐printed drug chamber was attached to the expandable layer using silicone adhesive.

### Fluid Flow Rate and Infused Volume Measurement

The flow rate of the fluid was analyzed by tracking 1 µm red fluorescent microspheres (R0100, Thermo Fisher Scientific) diluted in a 100:1 ratio with distilled water. To mitigate aggregation, the solution was sonicated for 5 min. The dynamic movement of the particles was observed and recorded using an inverted microscope (Eclipse Ti‐U, Nikon), along with a high‐speed digital camera (Phantom v7.3, Vision Research) and a fluorescent illuminator (Intenslight C‐HGFIE, Nikon) operating at 20,000 frames per second. The recorded video was then analyzed using Tracker (Open Source Physics) to determine the coordinates of the beads in each frame. After this analysis, the volumetric flow rate was computed by integrating the cross‐sectional area over the speed of the beads, followed by integrating the flow rate over time to determine the infused volume.

### Assembly and Disassembly Force Measurement

The external forces for assembling or disassembling the module (i.e., male adapter) and female adapter were measured using a force gauge (Series 5, Mark‐10). Given the magnetic attraction between the module and the female adaptor, no external force was required for assembly; we only measured the mutual attraction force between facing magnets to ensure their robust assembly.

Disassembly forces were measured in both horizontal (i.e., x and y) and vertical (i.e., z) directions, as defined in Figure 2B and Figure S14a (Supporting Information). Initially, the female adapter was bonded to an acrylic box, and a male module was assembled onto it. The acrylic box was aligned for the directional disassembly force measurement, as depicted in Figure S14c (Supporting Information), with a 1 mm overlap between the gauge and module. Horizontal disassembly forces were measured by descending the force gauge, with the process repeated for ±x and ±y directions, respectively. Vertical (i.e., +z) disassembly involved securing the male module to the gauge while the female adapter remained fixed to the floor and then raising the gauge.

### Characterization of Electrical Stimulation

The contact impedance of the electrical stimulation probe was measured using a potentiostat (PalmSens4, PalmSens) across a frequency range of 10 Hz—100 kHz, after immersing the probe in phosphate‐buffered saline (PBS; pH 7.4). Then, the same probe was connected to the optoelectronic module to evaluate its performance in modulating the intensity, frequency, and duty cycle of the electrical stimulation. The voltage applied to the stimulation probes inserted into the PBS was monitored using an oscilloscope (MSOX2004A, Keysight) while adjusting stimulation parameters. Notably, the intensity of electrical stimulation could be adjusted by switching resistors (0 Ω, 1.5 kΩ, 3.9 kΩ) connected to the signal output port (spring‐loaded pin).

### Animals

Adult (20—30 g) C57BL/6J mice, Thy1‐ChR2‐YFP, and Dbh‐IRES‐Cre mice were group‐housed on a 12‐h:12‐h light:dark cycle (lights on at 6:00 a.m.) with ad libitum access to food pellets and water. All mice in this study were transferred to a facility within the laboratory after weaning and remained in the facility in the laboratory at least 1 week before surgery, after surgery, and throughout the behavioral assays. All mouse procedures were approved by the Animal Care and Use Committee of Washington University (23‐0261) and conformed to U.S. National Institutes of Health (NIH) guidelines.

### Stereotaxic Device Implantation

Mice were anesthetized in an induction chamber (2–4% isoflurane) and placed into a stereotaxic frame (Kopf Instruments, Model 1900) to be maintained at 1–2% isoflurane. To test the optical stimulation module, Thy1‐Chr2‐YFP mice were implanted with µ‐ILED devices into the motor cortex (+1.0 mm (AP); ±0.5 mm (ML); and −0.5 (DV)) with implants secured and affixed with dental cement (C&B Metabond Adhesive Luting Cement, Parkell). To test the microfluidic module, Dbh‐IRES‐Cre were implanted unilaterally in Mice (−5.45 mm (AP); +0.65 mm (ML, with drug spout facing the midline); −4.25 mm (DV)). To test the electrofluidic modules, C57BL/6J mice were implanted in the right lateral hypothalamus (−1.2 (AP); +1.0 (ML); −5.0 (DV)). All mice were allowed to recover for at least 7 weeks following implantation before any behavioral testing.

### Drugs

The μ‐opioid receptor agonist, D‐Ala2, NMe‐Phe4, Glyol5‐enkephalin (DAMGO) (Research Biochemicals, Natick, MA) was dissolved in sterile 0.9% saline at a concentration of 0.25 µg/0.5 µL. The voltage‐gated sodium channel blocker, lidocaine hydrochloride monohydrate (Sigma–Aldrich), was dissolved in sterile artificial cerebral fluid at a concentration of 20mg mL^−1^ to make a 2% solution.

### General Behavioral Methods

For all in vivo experiments, mice were brought into a sound‐attenuated room maintained at 23 °C and allowed to acclimate for at least 30 min. Lighting was stabilized at ≈250 lux for all behaviors. Movements were video recorded via CCD camera and analyzed using Ethovision XT 13 (Noldus Information Technologies). Before each behavioral testing session, the appropriate MIND module was attached to each mouse.

### Home Cage Locomotor Assay

For the photostimulation, Thy1‐ChR2‐YFP mice (n  =  8) were placed into a new and clean home cage. Animals were allowed to explore the new cage for 10 min, after which a BLE signal was sent to the optoelectronic MIND module to drive 470 nm photostimulation at 20 Hz for 1 min followed by 1 min with no photostimulation, repeating over 8 min.

### Thermal Plantar Assay (Hargreaves Test)

To assess thermal nociception in mice, the Hargreaves test was used. Mice (n = 9) were acclimatized to the apparatus (IITC life science model 390G) for 30 min each day for 2 days without any exposure to thermal stimuli. On the test day, the mice were habituated to the Hargreaves equipment for 30 min. The light source intensity was set to 35%, ensuring that the thermal withdrawal latencies were ≈9–12 s. A pre‐determined cut‐off time of 20 s was used to prevent tissue damage. The light source was applied to both hind paws and measured the latency to evoke a withdrawal response. Two replicates were acquired per hind paw per mouse, and values for both paws were averaged at the end of the experiment. After the calculation of the baseline withdrawal threshold, DAMGO was infused using the MIND device via a BLE signal from the experimenter's phone. Mice were then retested and two new values were generated per hind paw.

### Open‐Field Locomotor Assay

For electrical stimulation, C57BL/6J mice (n = 8) were placed into a square (50 × 50 cm^2^) open‐field enclosure. In the first experiment, animals were allowed to explore the open‐field apparatus for 10 min, after which electrical stimulation (130 Hz, 2% duty cycle, and either 2.5 or 2.9 V were applied for 1 min. Mice were selected for the second experiment from those that increased their distance traveled (n = 6). For the second experiment, on a different day, were habituated to the open field for another 10 min and the same stimulation protocol from the first experiment was used. Then, mice that demonstrated at least a two‐fold increase in distance traveled were used for lidocaine infusion on the same day (n = 5). The experiment was then repeated and lidocaine was infused over 1 min and the OFF/ON stimulation paradigm was repeated.

### Tissue Processing

Animals were transcardially perfused with 0.1 m phosphate‐buffered saline (PBS) and then 40 mL 4% paraformaldehyde. Brains were dissected and post‐fixed in 4% paraformaldehyde overnight and then transferred to a 30% sucrose solution for cryoprotection. Brains were sectioned at 40 µm on a microtome and stored in a 0.01 m phosphate buffer at 4 °C before immunohistochemistry experiments.

### Immunohistochemistry

Mice were intracardially perfused with 4% paraformaldehyde, and then brains were sectioned (40 µm) and placed in 0.1 m phosphate buffer until immunohistochemistry. Free‐floating sections were washed in 0.1 m PBS for three 10 min intervals. Sections were then immersed in blocking buffer (0.5% Triton X‐100 and 5% natural goat serum in 0.1 m PBS) for 1 h at room temperature. Sections were then placed in primary antibody (rabbit anti cfos, 1:1000 or chicken anti‐tyrosine hydroxylase,1:2,000 Aves Labs) overnight at room temperature. After three 10 min washes in 0.1 m PBS, sections were incubated in secondary antibody (AlexaFluor 488 goat anti‐rabbit, or AlexaFluor 633 goat anti‐chicken, Life Technologies) for 2 h at room temperature, followed by another three 10 min washes in 0.1 m PBS. After immunostaining, sections were mounted and coverslipped with Vectashield HardSet mounting medium with DAPI (Vector Laboratories) and imaged on a Leica DM4 P epifluorescence microscope for image selection and then a Leica SP8 confocal microscope for final imaging.

### Data Presentation and Statistical Analysis

All values were given as individual data points and mean ± s.e.m. unless otherwise noted. Animal behavior data were analyzed for statistical significance by a two‐tailed *t*‐test as implemented in GraphPad Prism 9. Parametric tests were used when data was normally distributed.

## Conflict of Interest

The authors declare no conflict of interest.

## Author Contributions

I.K. and J.B. contributed equally to this work. I.K. and J.‐W.J. conceived the project and designed the detailed experimental protocols for overall technology development. I.K. and J.‐W.J. designed and fabricated the devices for animal experiments. I.K., J.E.Y., and J.‐W.J. designed the firmware and software. J.B., M.K.M., K.E.P., R.A., and J.G.M. designed the strategy for animal experiments. J.B., M.K.M., K.E.P., N.D.L.K., R.A., and J.G.M. performed the animal experiments. C.S., J.‐S.Y., J.‐B.Y., and J.X. performed the simulations. I.K., J.B., M.K.M., K.E.P., B.‐J.J., J.L., N.D.L.K., W.L., J.G.M., and J.‐W.J. performed the investigation and analyzed the data. I. K., J.B., C.Y.K., D.A.K., M.K.M., K.E.P., J.G.M., J.‐W.J. wrote the paper. J.B., R.A., J.G.M., and J.‐W.J. acquired funding and supervised the project. J.G.M. and J.‐W.J. are co‐senior authors. All authors discussed the results and contributed to the revision of the manuscript.

## Supporting information



Supporting Information

Supplemental Movie 1

## Data Availability

The data that support the findings of this study are available from the corresponding author upon reasonable request.
