# Supplementary material for: Wireless Modular Implantable Neural Device with One‐touch Magnetic Assembly for Versatile Neuromodulation
Source: Adv Sci (Weinh). 2024 Dec 3;12(4):2406576. doi: 10.1002/advs.202406576 (PMC11775568; doi:10.1002/advs.202406576)
Supplement: Supplementary file 1 — Supporting Information [file ADVS-12-2406576-s002.docx]

## Supporting Information for

**Wireless modular implantable neural device with one-touch magnetic assembly for versatile neuromodulation**

Inho Kang^1†^, John Bilbily^2,3,4,5,6†^, Choong Yeon Kim^1,7^, Chuanqian Shi^8^, Manish K. Madasu^2,3,4,5^, Eun Young Jeong^1^, Kyle E. Parker^2,3,4,5^, Do A Kwon^1,9^, Bum-Joon Jung^10^, Jae-Soon Yang^1^, Juhyun Lee^1^, Noah D.L. Kabbaj^2,3,4,5^, Wonhee Lee^10,11,12^, Jun-Bo Yoon^1^, Ream Al-Hasani^2,3,4,5^, Jianliang Xiao^8^, Jordan G. McCall^2,3,4,5*^, Jae-Woong Jeong^1,13*^

†These authors contributed equally to this work.

*To whom correspondence should be addressed. E-mail: jjeong1@kaist.ac.kr (J.-W.J.); jordangmccall@wustl.edu (J.G.M.)

**This supplementary file includes:**

Notes S1-S2

Figures S1-S14

Tables S1-S2

**Other supplementary material for this manuscript includes the following:**

Movie S1

**Supplementary Note**

**Note S1. Engineering disassembly: A pillar-based approach for mitigating stress in the magnetic assembly of MIND**

Stable fluidic and electrical connections in MIND are ensured through magnetic assembly. To prevent excessive stress induced on animals during disassembly, MIND is designed to weaken the disassembly force by aligning the module in the orthogonal direction of magnetic attraction (i.e., horizontal direction). However, to avoid unintended disassembly during animal behavior experiments, we introduced a pillar structure.

Each module contains two pillar structures, akin to Lego bricks, and is inserted into female adapters. Externally applied horizontal forces drive disassembly through rotation, utilizing mutual magnetic attraction and inserted column structures. One side of the module where the external force applied (left side of the magnetic assembly in Supplementary Fig. 6a) lifts while the opposite side (right side of the magnetic assembly (P1) in Supplementary Fig. 6a) maintains contact with the female adapter, acting as the axis of rotation.

Assuming that the axis of rotation remains in contact during disassembly, the condition required for disassembly can be expressed as

$r= \sqrt{{(a+d_{2})}^{2}+h^{2}}$ , (S1)

and the only variable, $a$, which changes during the disassembly process can be expressed as follows:

$a=w\times\cos\left( \tan^{-1} \left( \frac{h}{a+d_{2}} \right) \right)$, (S2)

where $a$ is the distance from the axis of rotation to the pillar, *w* is the thickness of the female adapter wall containing the axis of rotation, *h* is the length of the pillar, *d_1_* is the diameter of the hole of the female adapter, *d_2_* is the diameter of the pillar, and *r* is the rotational radius.

To ensure smooth disassembly without any obstruction, the sum of the thickness of the female adapter wall (*w*) and the diameter of the hole (*d_1_*) need to be longer than the rotational radius (*r*). This engineering consideration guarantees that the rotating module can be freely disassembled without getting stuck, as the sufficient clearance provided by *w*+*d_1_* > *r* allows the pillar to rotate out from the hole smoothly. By engineering the distance between the axis of rotation and the nearest pillar, MIND is configured to disassemble exclusively in the -x direction as defined in Fig.2b, thereby optimizing its performance and suability during the disassembly process.

**Note S2. Critical leaking pressure of MIND’s fluidic connection**

The critical condition for leakage resulting from elastic deformation in Neo-Hookean material is described by the leak pressure formula:

$P_{c}=\mu\frac{L}{H}\sqrt{6\left( \left( 1+\varepsilon_{z} \right)^{-4}-1 \right)}$ (S3)

where *P_c_* is the critical pressure of fluid leaking; *μ* is the shear modulus of PDMS; *L* indicates the difference between the outer radius (*R*) and inner radius (*r*) of the gasket; *H* is the thickness of the gasket; ε_z_ is the normal strain at the bottom surface of the gasket which was calculated by finite element analysis (FEA). Here, *μ =*0.67 MPa; *L = R – r =* 0.4 mm; H = 0.6 mm, and PDMS was set as a Neo-Hookean material.

The drug cartridge is inserted into the female adapter in the +z direction. The top side of the PDMS gasket is fixed to the drug cartridge pillar, while the bottom surface is compressed by magnetic force and makes contacts with the female adapter (Supplementary Fig. 8a). According to the FEA result, the normal strain on the bottom surface along the +z direction (*ε_z_*) ranges from -31.99% to -6.269%, and the critical leaking pressure (*P_c_*) ranges from 0.592 to 2.1 MPa. Thus, the lowest leaking pressure is ~592 kPa, significantly higher than the fluid pressure during drug delivery (Supplementary Fig. 8b).

**Supplementary Figures**

**
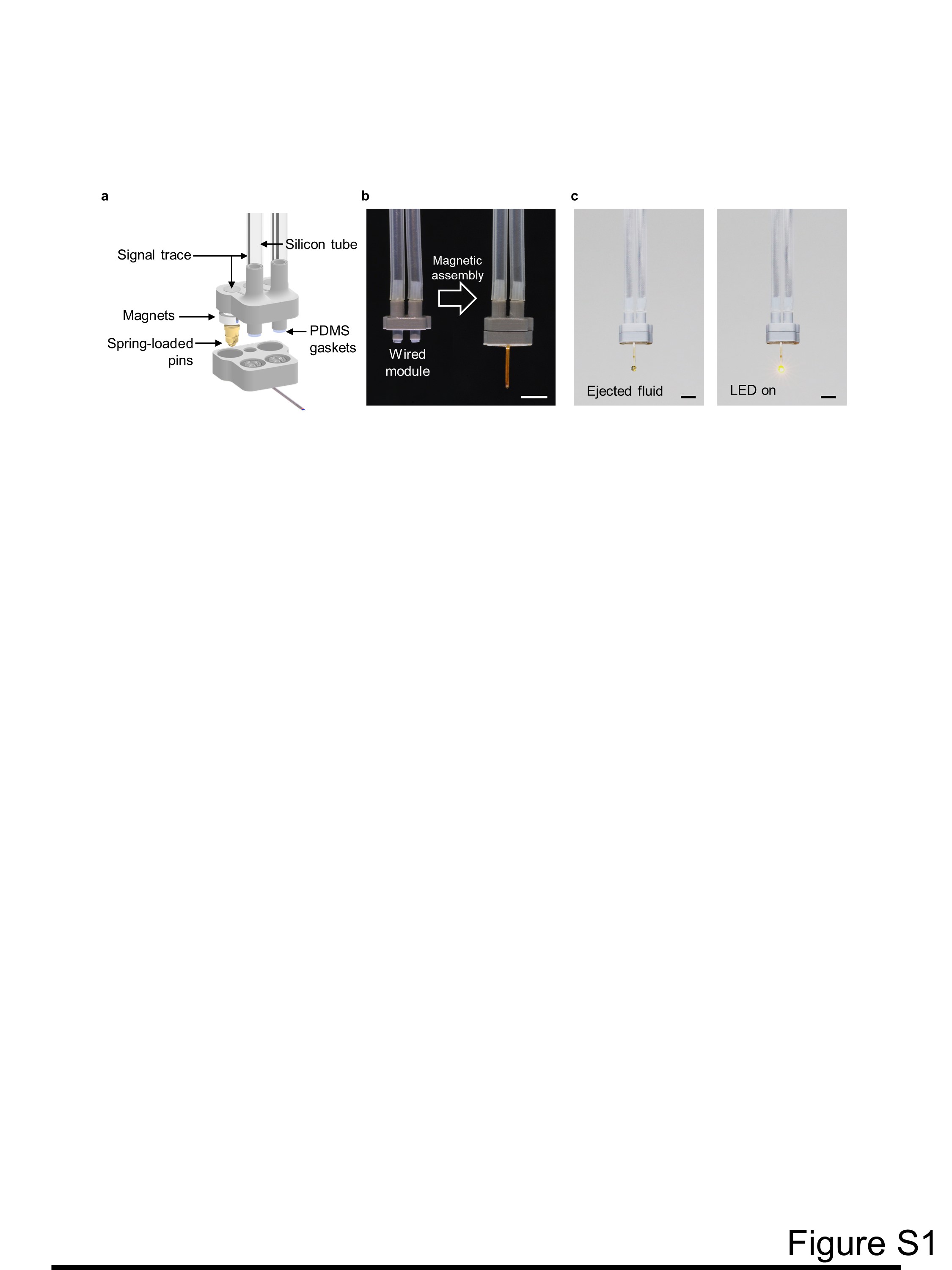
**

**Figure S1 | Design and functionality of the wired module with optofluidic probe. a**. Exploded schematic view of the wired module. **b**, One-touch magnetic assembly of the wired module (Scale bar, 5 mm). **c**, Optical images demonstrating drug delivery (left) and photostimulation (right) of the wired module (Scale bars, 5 mm).

**
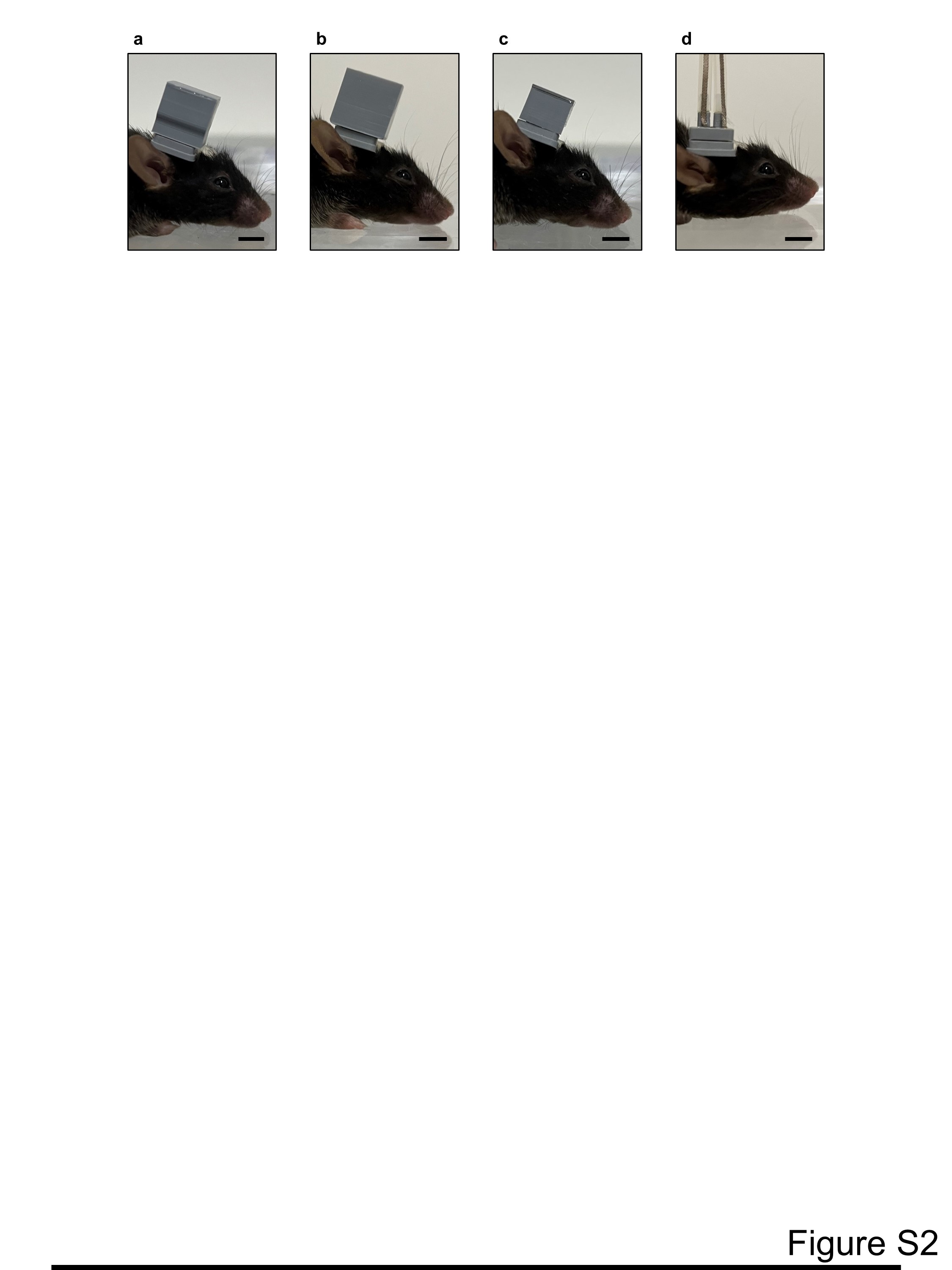
**

**Figure S2 | Replacement of four different modules on a single implanted neural probe. a-d**, Optical images depict the assembly process of the optofluidic module (**a**), multidose module (**b**), optoelectronic module (**c**), and wired modules (**d**) (Scale bars, 5 mm).

**
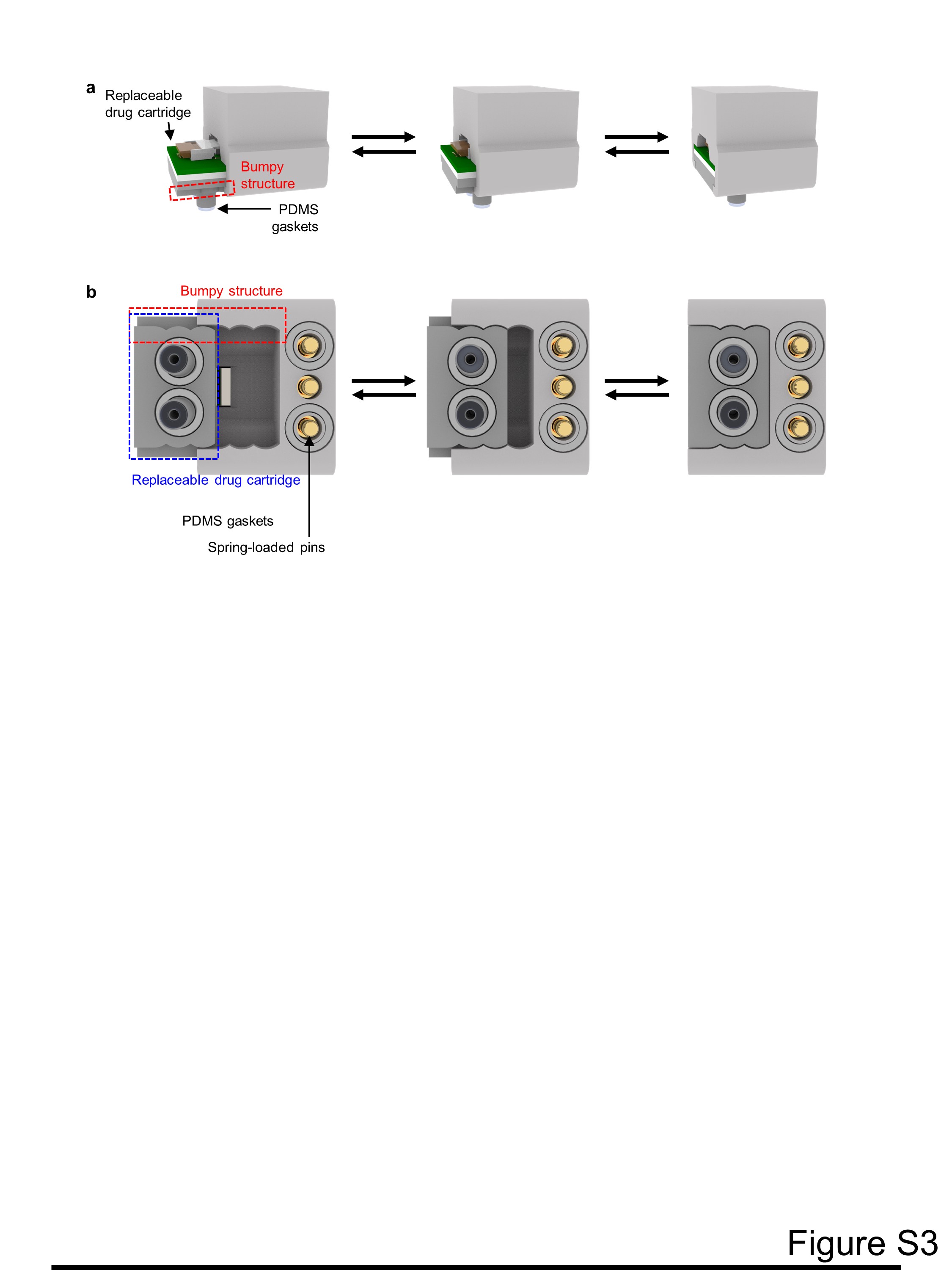
**

**Figure S3 | Assembly and disassembly of a drug cartridge using frictional forces of its bumpy structure. a**, Side view illustrating the drug cartridge replacement process. **b**, Bottom view highlighting a pair of consecutive bumpy structures for secure assembly.

**
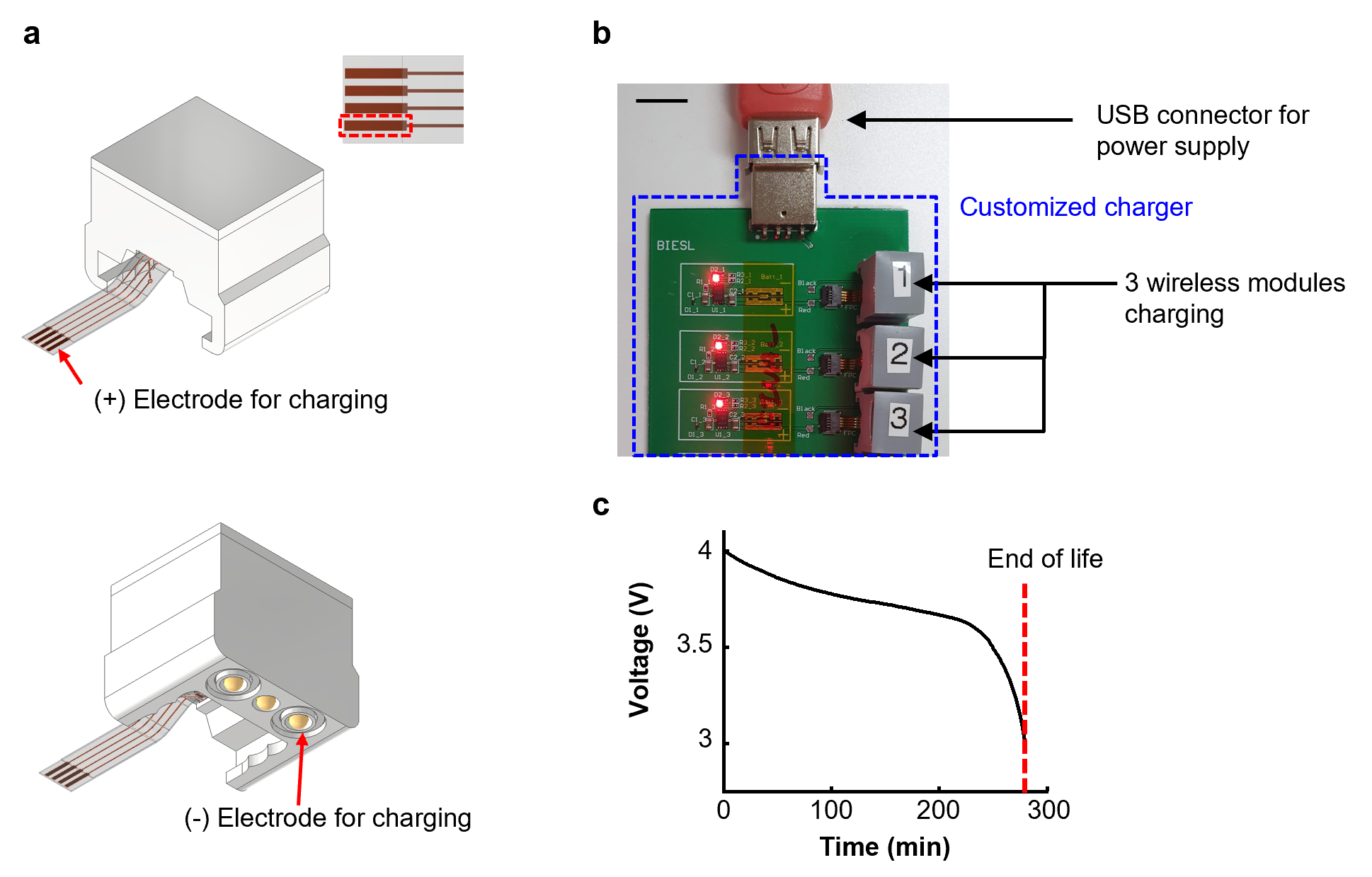
**

**Figure S4 | Battery charging using one-touch magnetic assembly of modules. a**, Schematic illustrations depicting the charging electrodes of a wireless module. The positive and negative electrodes of the battery are, respectively, an electrode from the FPC cable and a spring-loaded pin, as indicated in the figure. **b**, Optical image demonstrating the simultaneous recharging of multiple modules using the customized charger. Integrated batteries in the modules are charged by simply placing the modules on the magnetic charging electrodes of the charger with the FPC cable plugged in. Scale bar, 10 mm. **c**, battery discharge profile of the optoelectronic module during optogenetic stimulation (470 nm wavelength, 20 Hz with 10 ms pulse width) after completing the actuation of two chambers at t = 0 min.

**
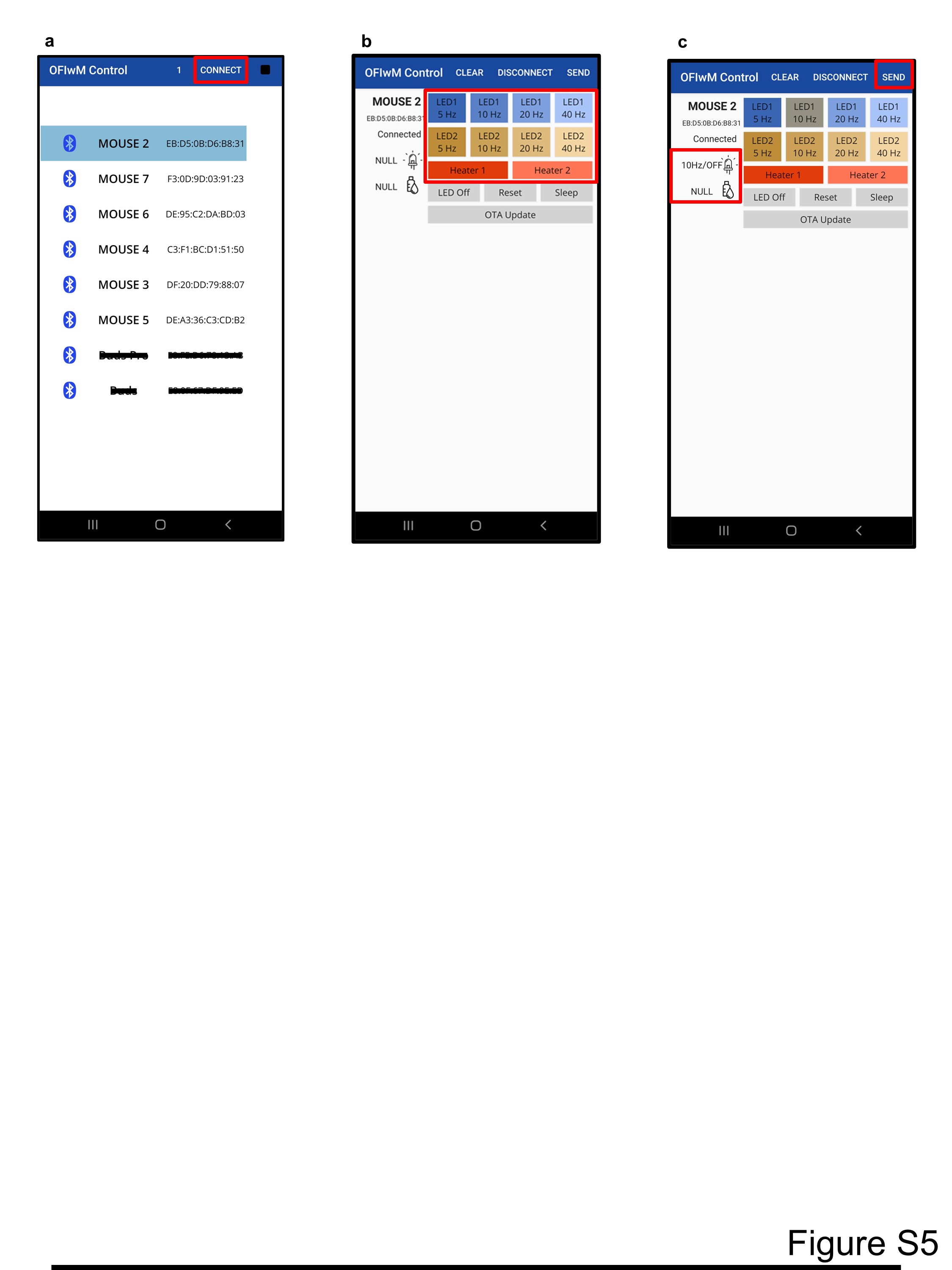
**

**Figure S5 | Smartphone application for MIND. a**, Device selection and connection page. **b**, Neuromodulation parameter setting. **c**, Stimulation command transmission and current status monitoring.

**
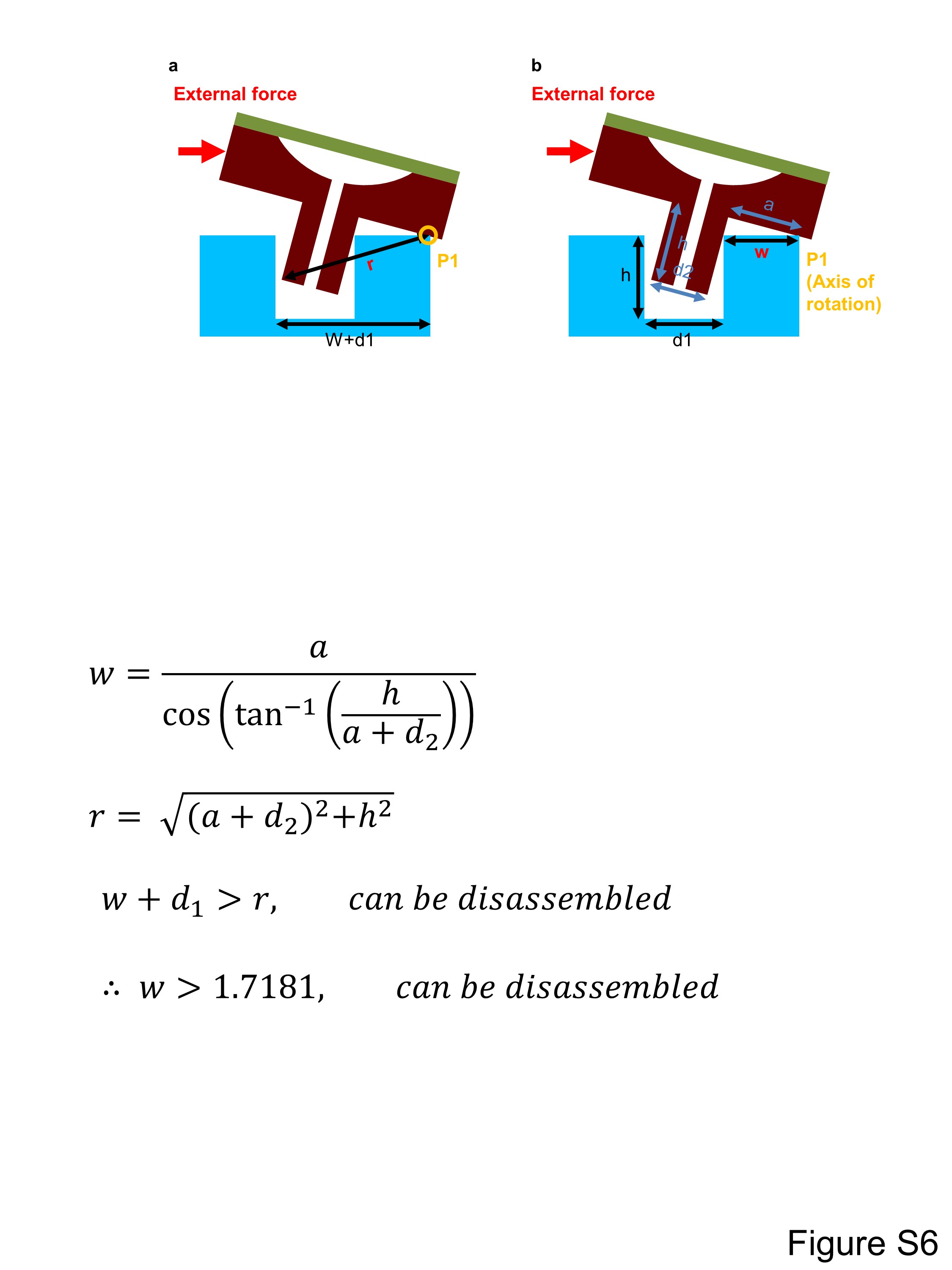
**

**Figure S6 | Rotational disassembly by lateral external force. a**, Schematic diagram describing rotational radius for disassembly. **b**, Schematic diagram defining variables.

**
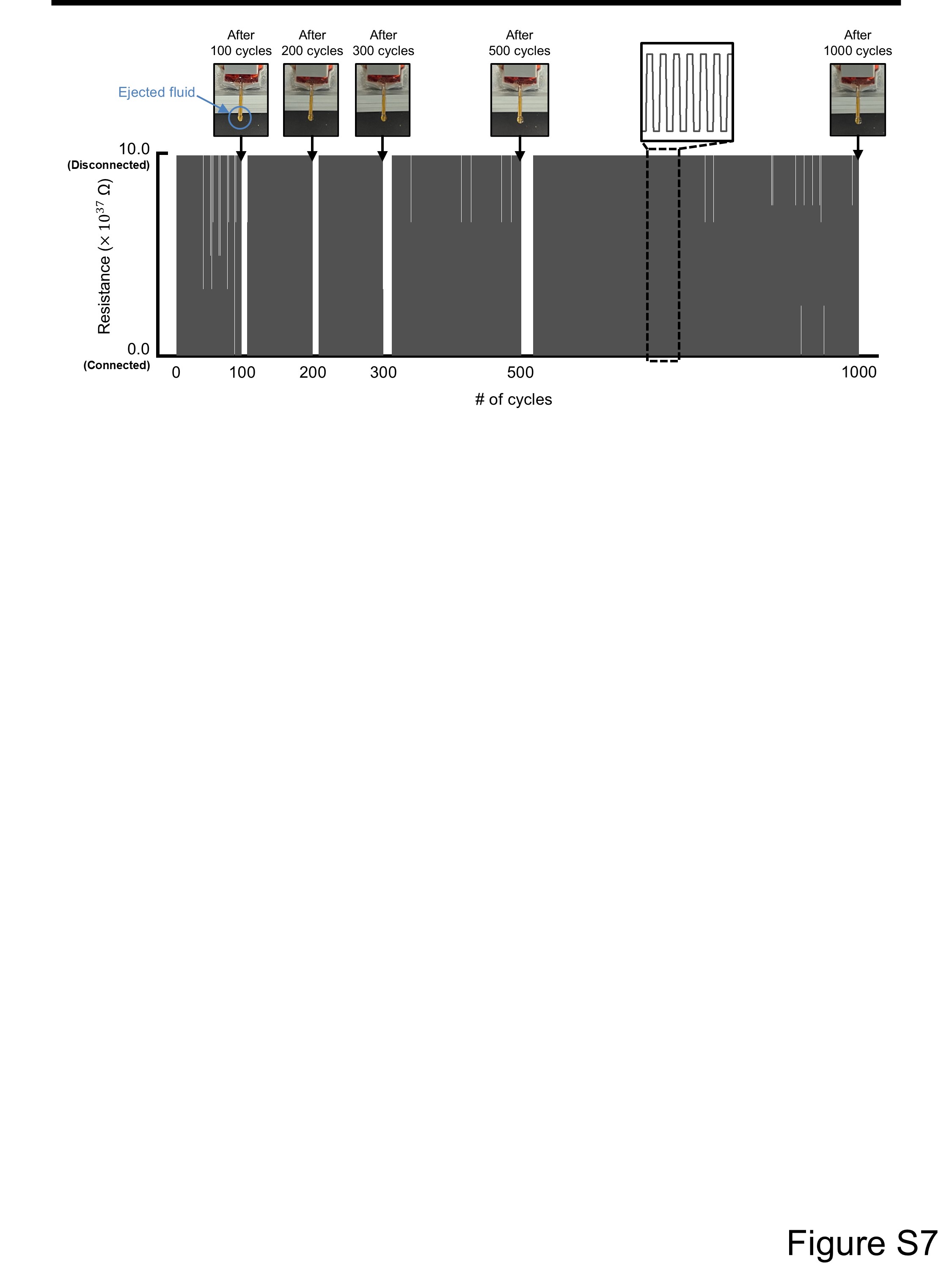
**

**Figure S7 | Reliability test results for the MIND device connections.** The graph shows the electrical resistance of the MIND’s electrical connections over 1000 consecutive cycles of assembly and disassembly, with consistent pulse train patterns indicating reliable electrical connections throughout the test. Fluidic connectivity was assessed after specific intervals (100, 200, 300, 500, and 1000 cycles), as illustrated in the inset images, where fluid ejection confirms the maintenance of a stable fluidic connection. These results demonstrate the reliability of the MIND device in sustaining both electrical and fluidic connections during prolonged use.

**
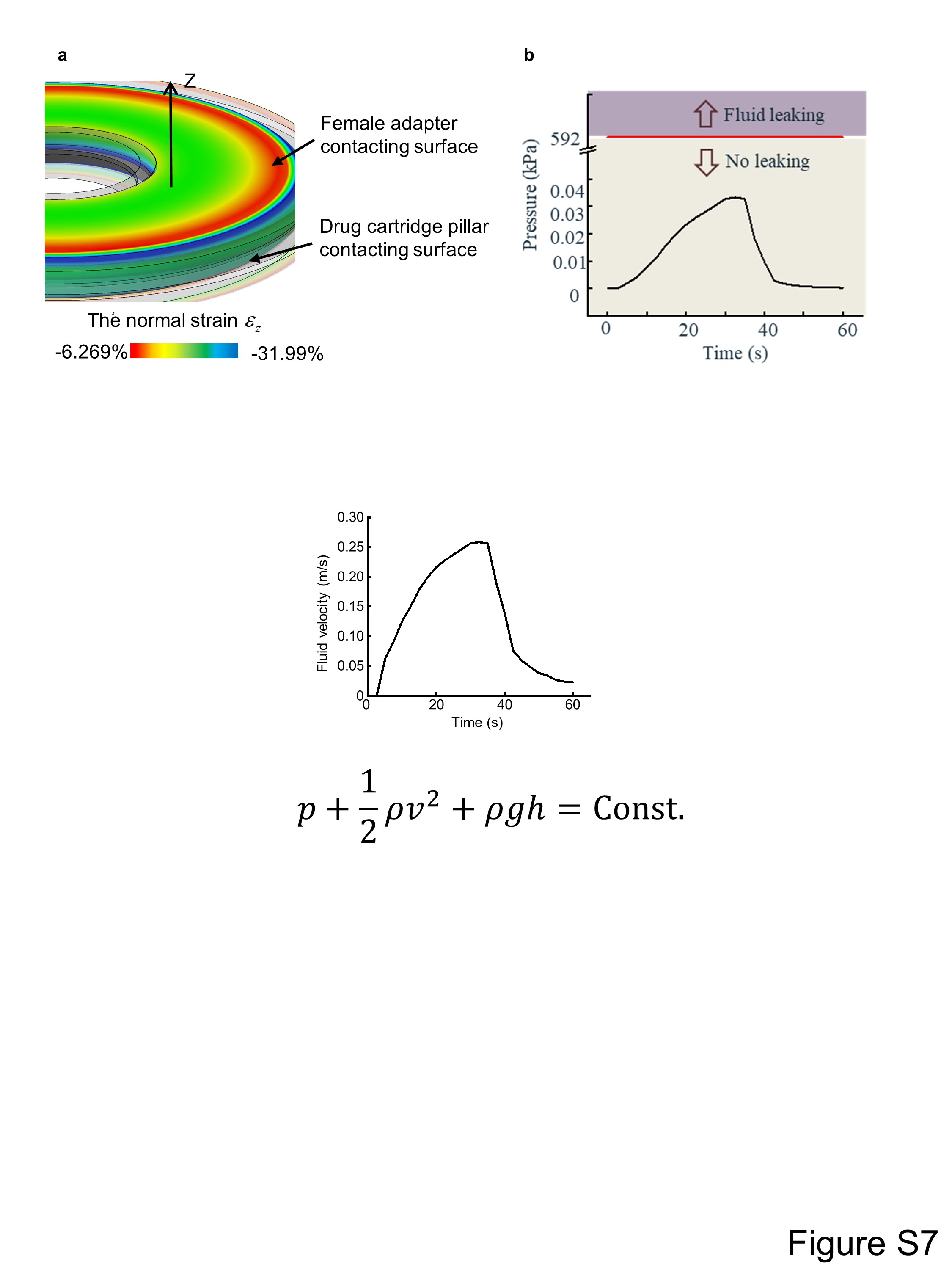
**

**Figure S8 | FEA analysis of the critical leaking pressure of MIND’s fluidic connection. a**, Finite element analysis (FEA) simulation showing distributed normal strain on a PDMS gasket. **b**, Fluid leaking pressure and internal chamber pressure as a function of time during actuation.

**
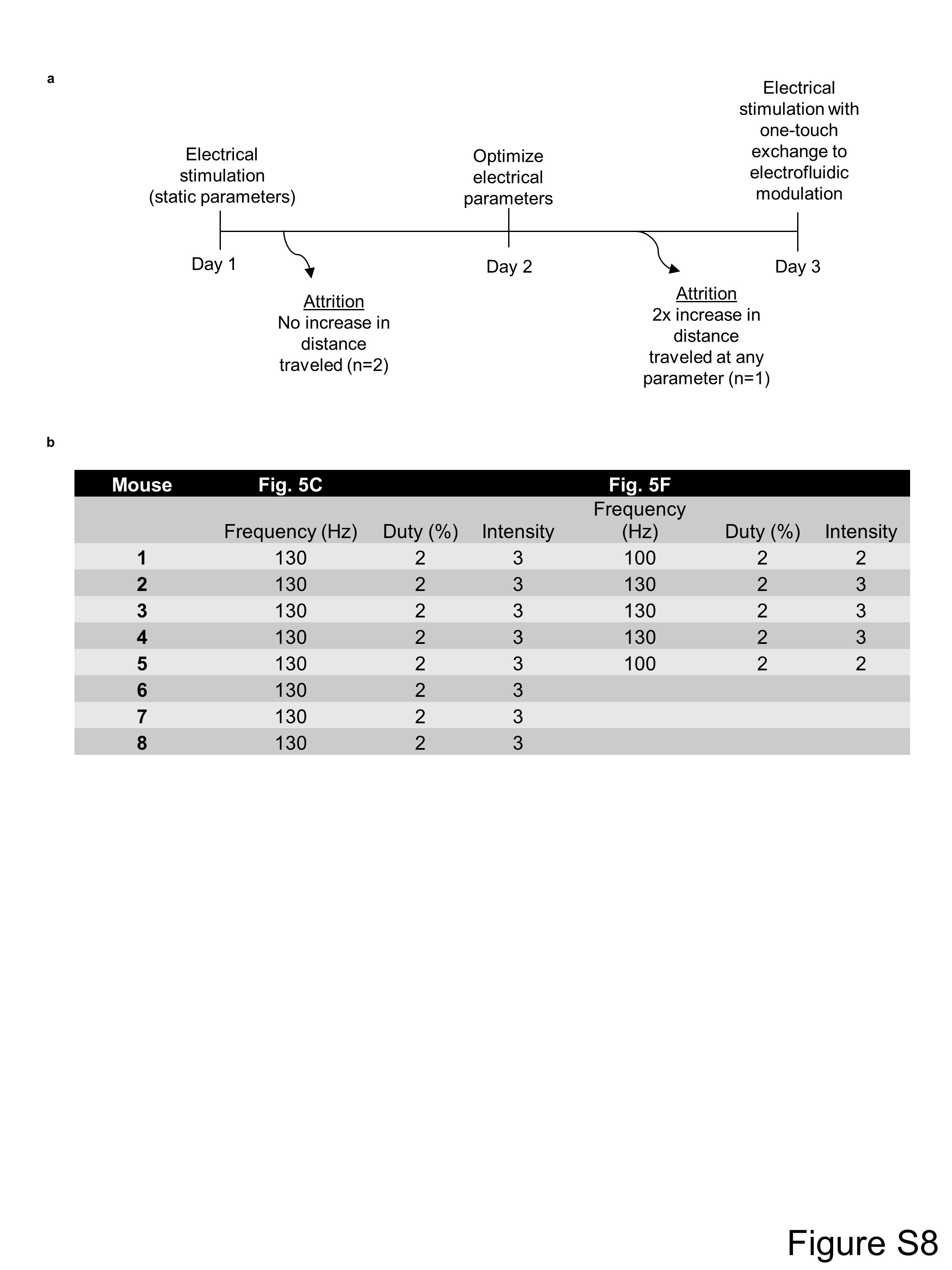
**

**Figure S9 | Experimental timeline and parameter adjustments for electrical stimulation. a**, Timeline: Day 1 (static parameters, no distance increase, n=2), Day 2 (optimization), Day 3 (one-touch exchange, 2x distance increase, n=1). **b**, Parameter settings for each mouse (Frequency, Duty cycle, Intensity) used in Fig. 5C and Fig. 5F.

**
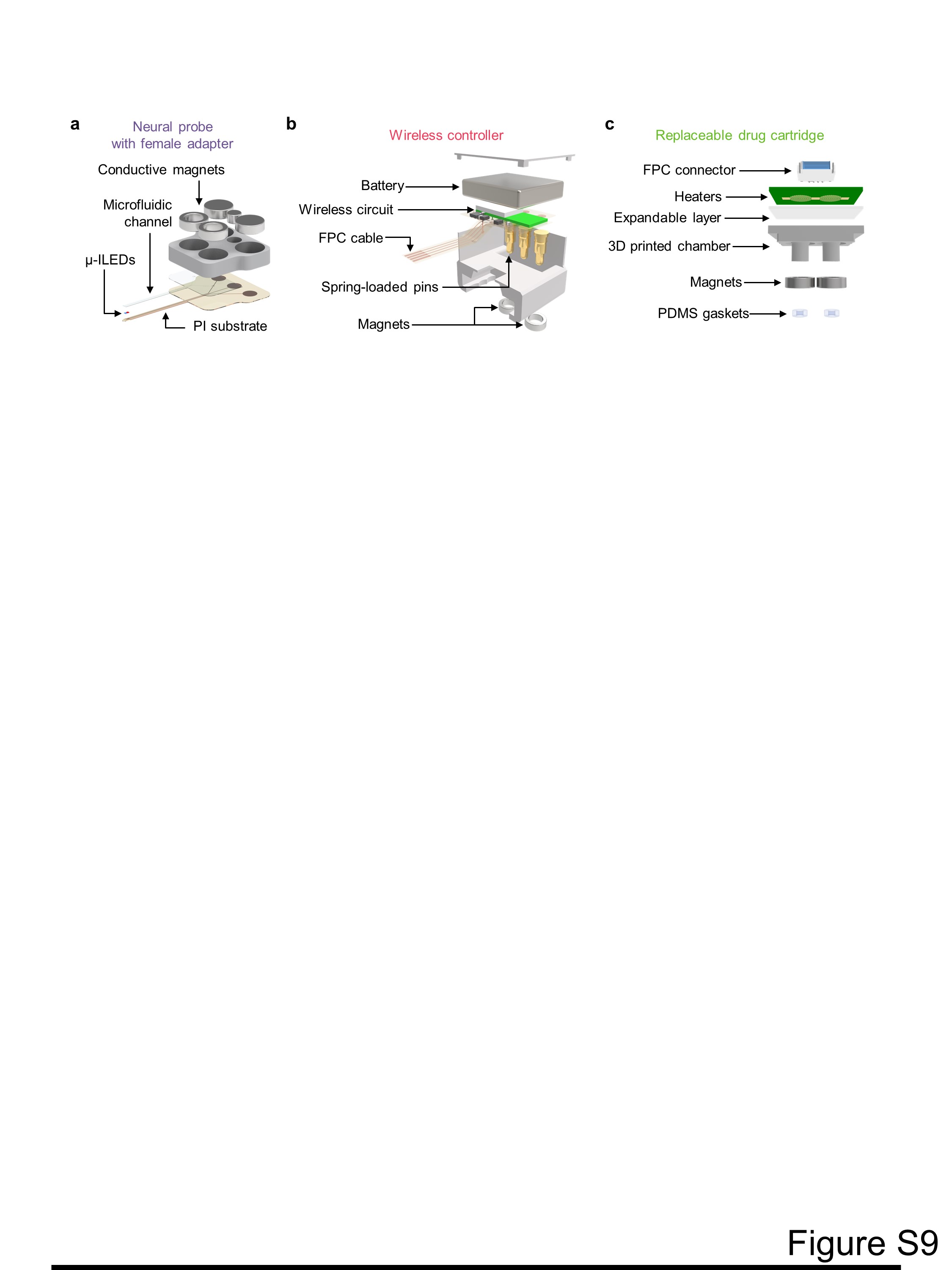
**

**Figure S10 | Constituent parts of MIND. a-c,** Exploded schematic views of neural probe with female adapter (**a**)**,** wireless controller (**b**), and replaceable drug cartridge (**c**).

**
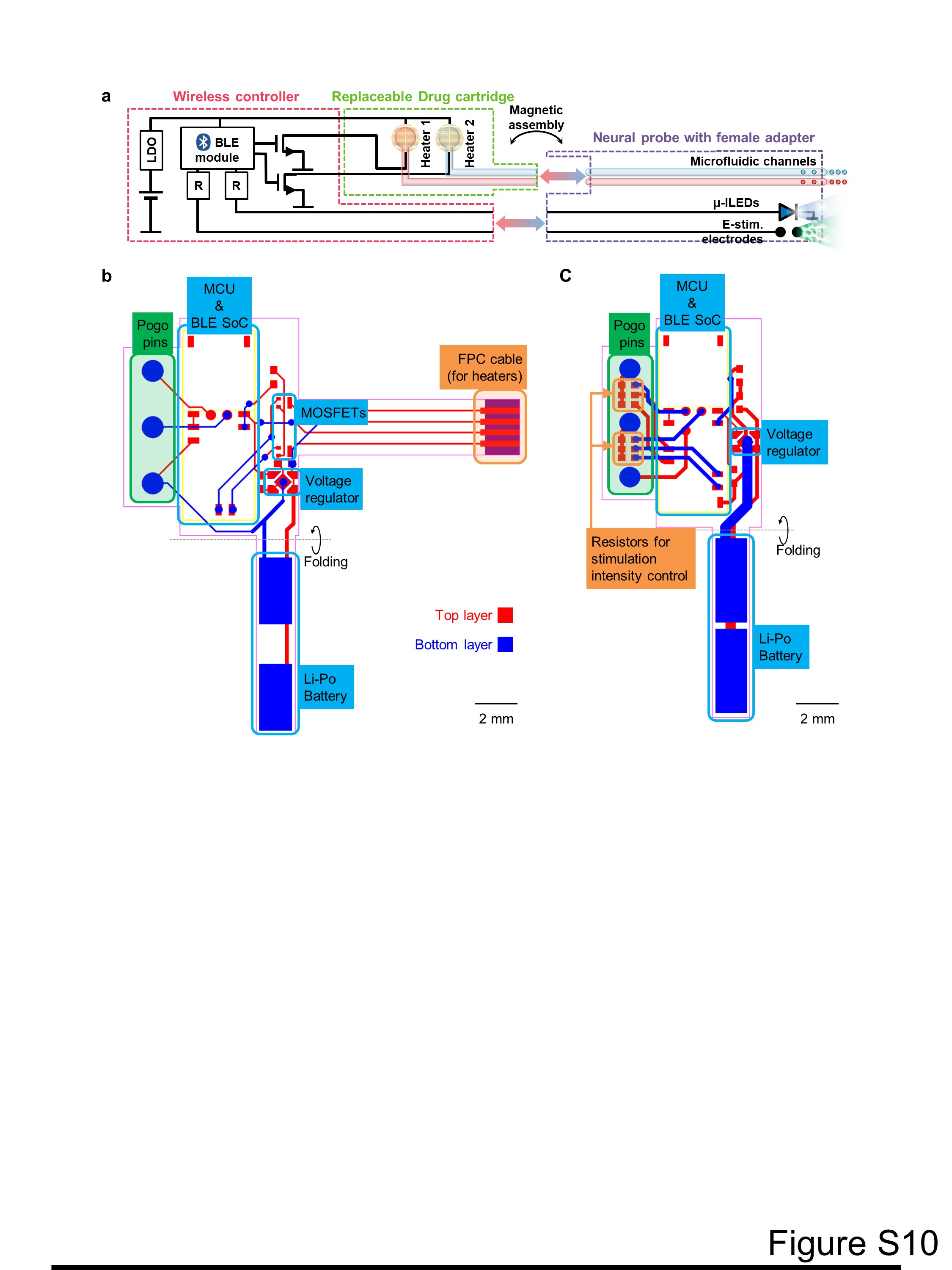
**

**Figure S11 | MIND circuit design. a**, Schematic circuit diagram of MIND. **b**, PCB schematic of the optofluidic module capable of optical stimulation, and fluid delivery. **c**, PCB schematic of the optoelectronic module capable of generating electrical and optical stimulation signals.


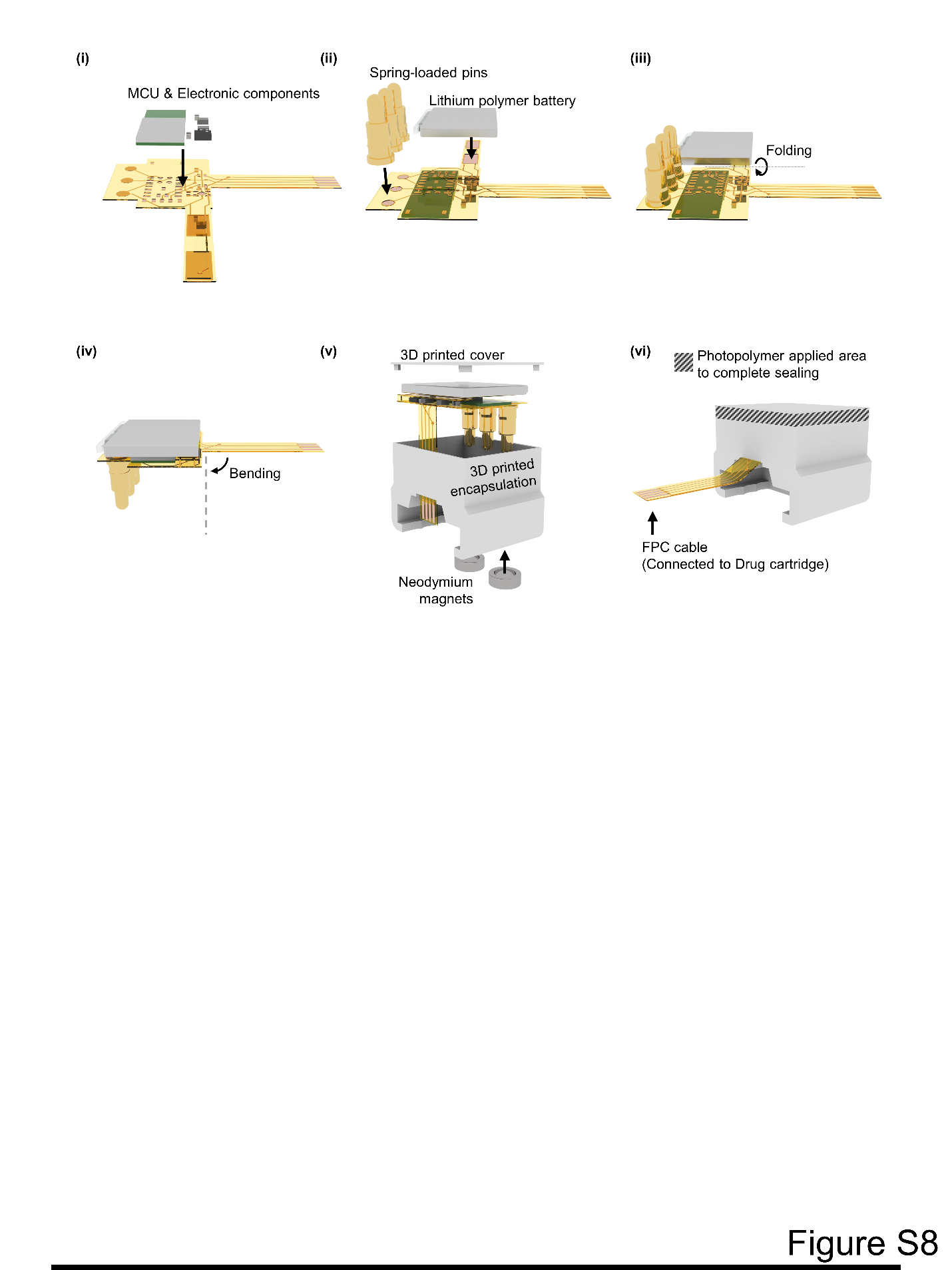


**Figure S12 | Manufacturing process of MIND.** (i) Soldering of electronic components, including the BLE SoC, onto the top layer of a flexible PCB (0.1 mm-thick; PI substrate). (ii) Mounting spring-loaded pins and a battery on the bottom layer of the PCB. (iii) Folding the wing attached to the battery. (iv) Bend the exposed FPC cable at a 90-degree angle. (v) Inserting the folded circuit into a 3D printed encapsulation with magnets embedded at the bottom. (vi) Sealing it with a 3D printed cover to complete the fabrication.

**
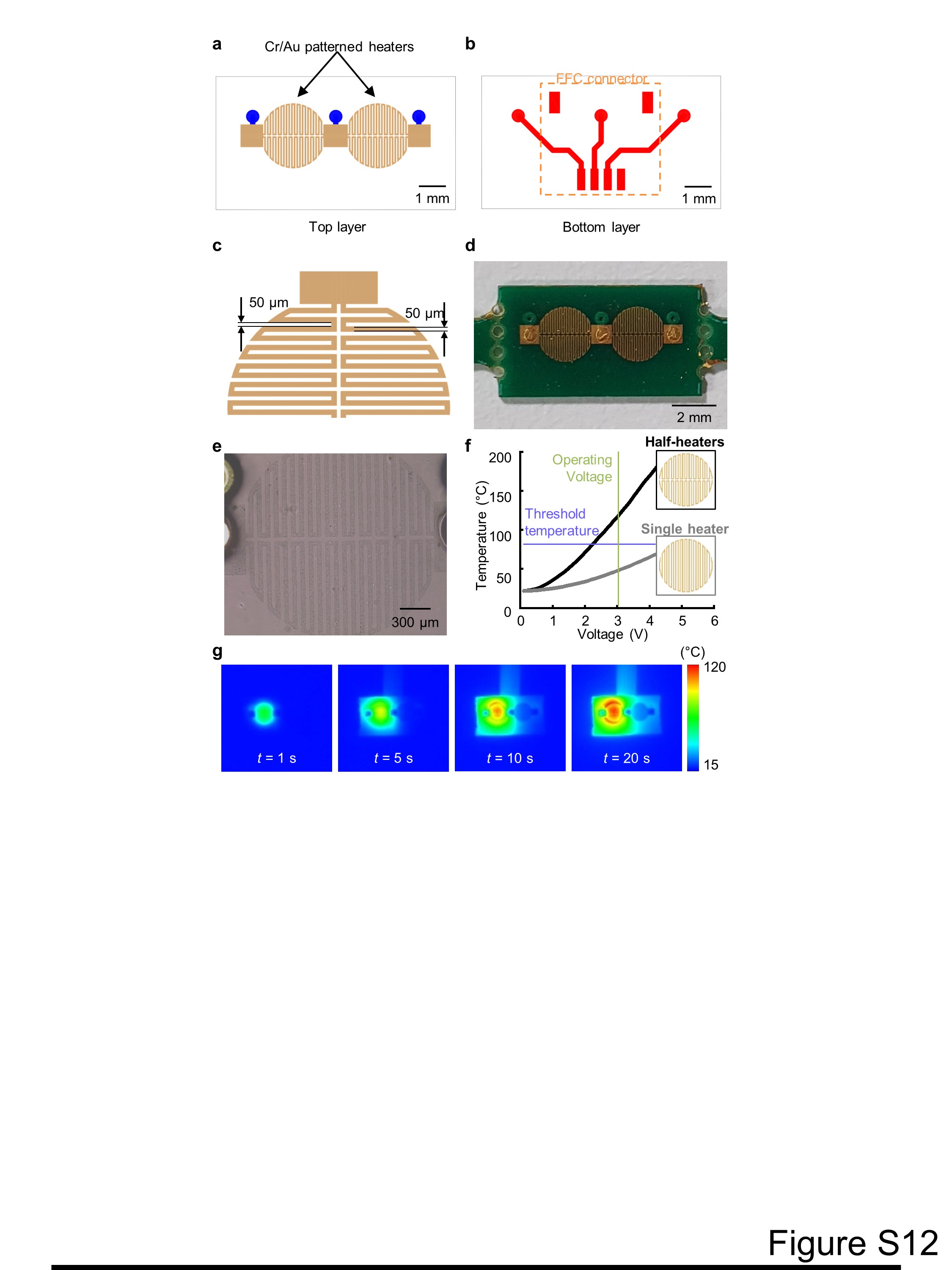
**

**Figure S13 | Thermal actuator of MIND. a-b**, Illustrations of top layer (microheater; **a**) and bottom layer (interconnects; **b**) of the thermal actuator. **c**, Illustration depicting the dimensions of the metal traces and gaps in the thermal actuator. **d**, Optical image of the fabricated heaters. **e**, Microscopic image of the patterned photoresist for the gold lift-off process. **f**, Comparison of the temperatures induced by thermal actuators with two parallel-connected half heaters and a single heater as a function of voltage. **g**, Sequential thermal images of the actuator captured with an IR camera when actuation was initiated at t = 0 s.

**
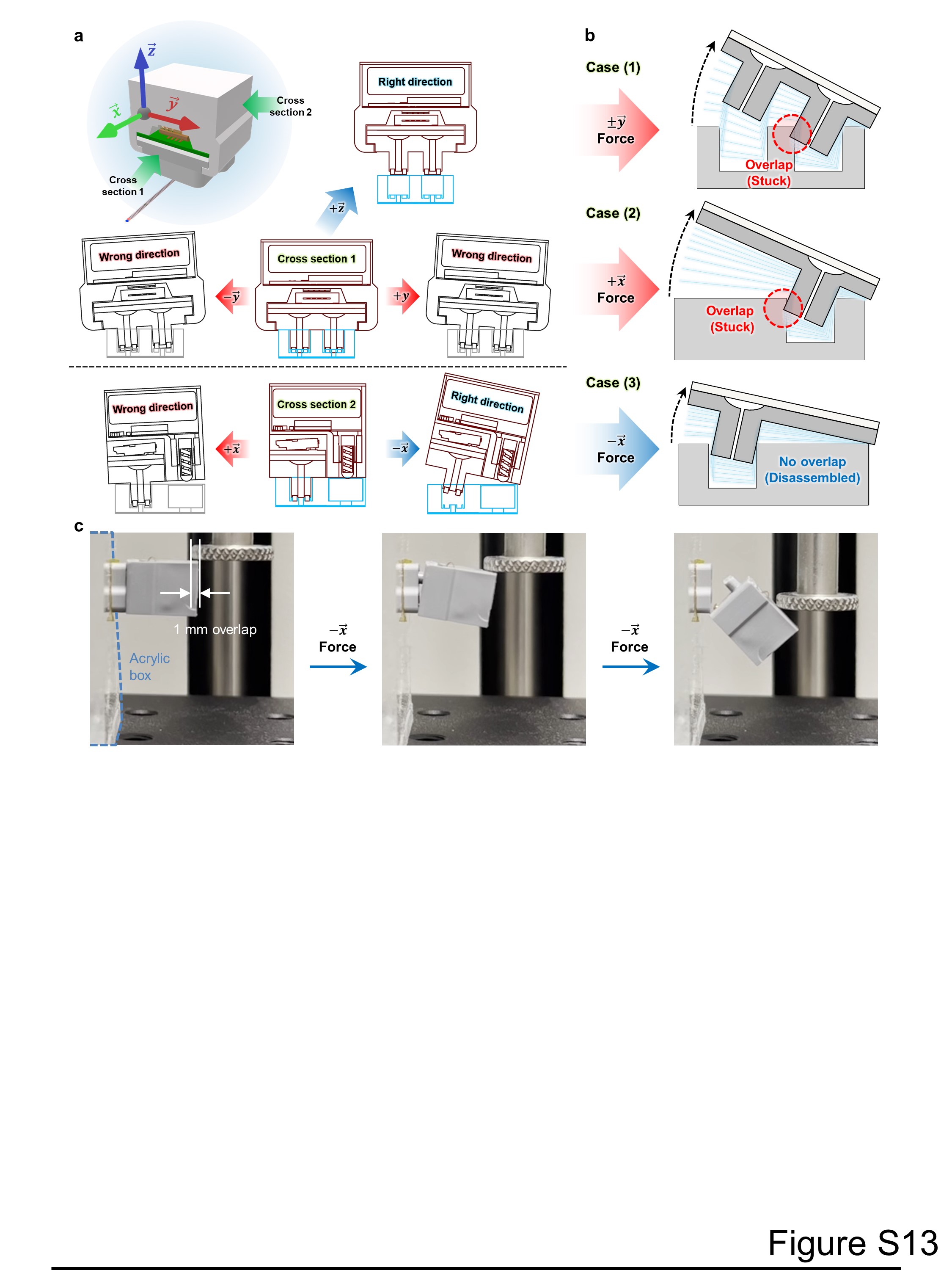
**

**Figure S14 | Analysis of decomposability of magnetically assembled MIND in each direction. a**, Schematic illustrations defining each axis direction and confirming the decomposability in each direction. In the vertical direction (+z), device is disassembled by overcoming the attraction of permanent magnets. When external force is applied in the horizontal direction, MIND is disassembled only in the -x direction, not in the ±y directions and +x direction. **b**, Illustrations depicting the rotational movement of assembled module when a horizontal external force is applied. MIND remains assembled when the external force is applied in the ±y directions and +x direction since the pillar structures inserted into the female adapter become stuck (top and middle), while the pillar structures do not become stuck when the force is applied in the –x direction due to the larger rotational radius (bottom). **c**, Sequential optical images demonstrating disassembly in the -x direction using a force gauge.

**Supplementary Table**

**Table S1. Comparison of the state-of-the-art multimodal neural implants**^[12,13,16,17,19]^


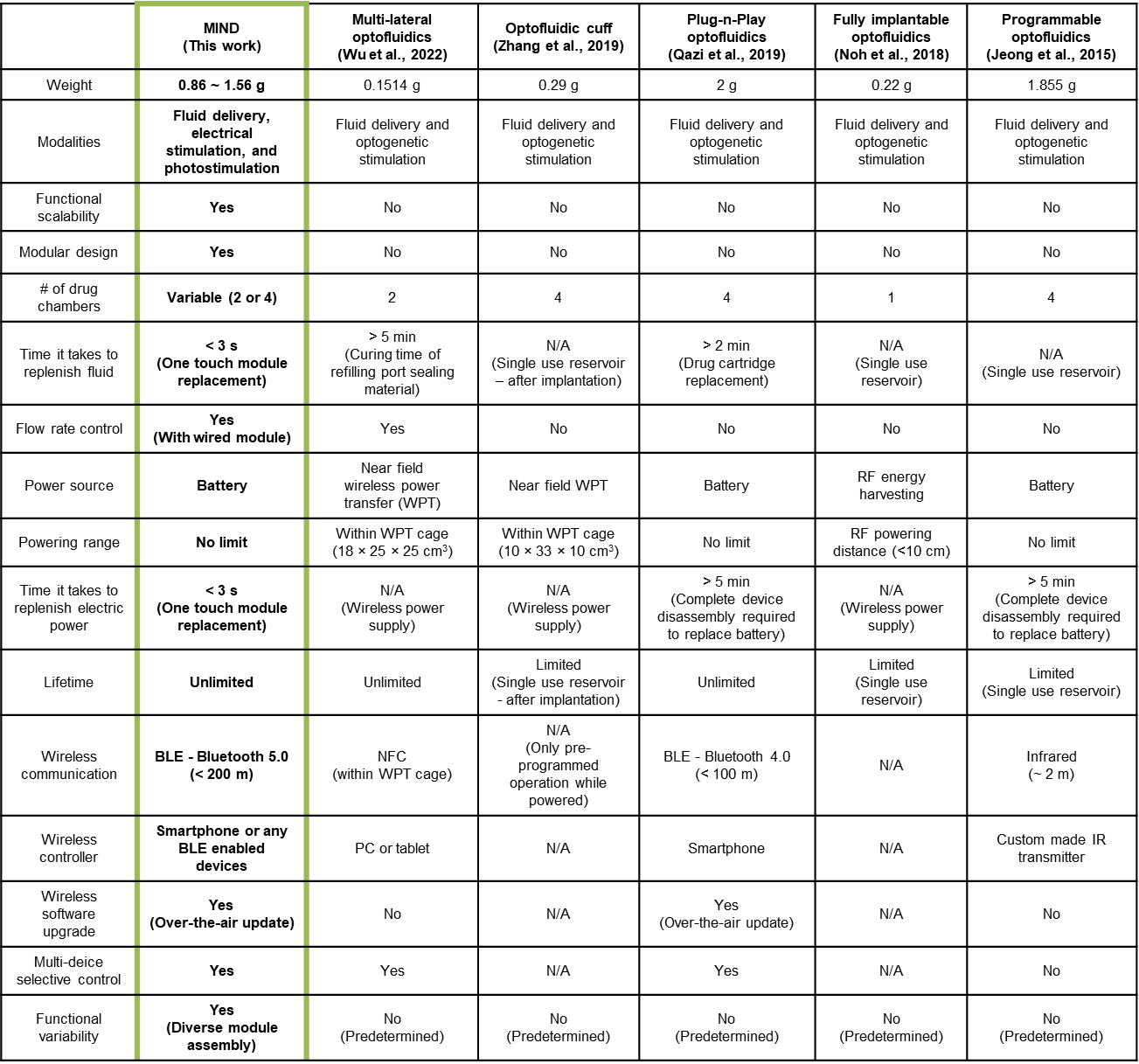


* Bold texts highlight the strengths of our work.

**Table S2. Detailed specifications of developed modules**

|  | **Optofluidic**  **module**  **(Fig. 1B(i))** | **Electrofluidic**  **Module**  **(Fig. 1B(i))** | **Multidose module**  **(Fig. 1B(ii))** | **Optoelectronic module**  **(Fig. 1B(iii))** | **Wired module**  **(Fig. 1B(iv))** |
| --- | --- | --- | --- | --- | --- |
| **Weight** | 1.56 g | 1.56 g | 1.30 g | 0.86 g | 0.34 g  (excluding wires) |
| **Volume** | 1050 mm^3^ | 1050 mm^3^ | 1020 mm^3^ | 670 mm^3^ | 230 mm^3^  (excluding wires) |
| **Capabilities** | - Optical stimulation  - Fluid delivery | - Electrical stimulation  - Fluid delivery | - Fluid delivery | - Optical stimulation  - Electrical stimulation | - Optical stimulation  - Electrical stimulation  - Fluid delivery |
| **# of signal channels** | 2 (for optical stimulation) | 2 (for electrical stimulation) | N/A | 2 (for optical and/or electrical stimulation) | 2 (for optical and/or electrical stimulation) |
| **# of drug chambers** | 2 (individual chambers) | 2 (individual chambers) | 4 (sequentially connected chambers) | N/A | N/A |
| **# of fluidic channels** | 2 | 2 | 1 | N/A | 2 |

**Supplementary Movie**

**Movie S1. Movie of MIND’s multimodal neuromodulation with one-touch replacement.**
